# The OsEIL1‐OsERF115‐target gene regulatory module controls grain size and weight in rice

**DOI:** 10.1111/pbi.13825

**Published:** 2022-05-06

**Authors:** Chang Liu, Tian Ma, Dingyang Yuan, Yang Zhou, Yan Long, Ziwen Li, Zhenying Dong, Meijuan Duan, Dong Yu, Yizhi Jing, Xiaoyue Bai, Yanbo Wang, Quancan Hou, Shuangshuang Liu, Jin‐Song Zhang, Shou‐Yi Chen, Dayong Li, Xue Liu, Zhikang Li, Wensheng Wang, Jinping Li, Xun Wei, Biao Ma, Xiangyuan Wan

**Affiliations:** ^1^ 12507 Shunde Graduate School Research Center of Biology and Agriculture Zhongzhi International Institute of Agricultural Biosciences University of Science and Technology Beijing Beijing China; ^2^ Beijing Engineering Laboratory of Main Crop Bio‐Tech Breeding Beijing International Science and Technology Cooperation Base of Bio‐Tech Breeding Beijing Solidwill Sci‐Tech Co. Ltd. Beijing China; ^3^ Guangdong Laboratory for Lingnan Modern Agriculture College of Agriculture South China Agricultural University Guangzhou China; ^4^ State Key Laboratory of Hybrid Rice Hunan Hybrid Rice Research Centre Changsha China; ^5^ College of Agronomy Hunan Agricultural University Changsha China; ^6^ State Key Laboratory of Plant Genomics Institute of Genetics and Developmental Biology Chinese Academy of Sciences Beijing China; ^7^ National Engineering Research Center for Vegetables Beijing Vegetable Research Center Beijing Academy of Agriculture and Forestry Science Beijing China; ^8^ Institute of Crop Sciences Chinese Academy of Agricultural Sciences Beijing China

**Keywords:** grain size and weight, *OsEIL1*, *OsERF115*, regulatory module, rice

## Abstract

Grain size is one of the essential determinants of rice yield. Our previous studies revealed that ethylene plays an important role in grain‐size control; however, the precise mechanism remains to be determined. Here, we report that the ethylene response factor OsERF115 functions as a key downstream regulator for ethylene‐mediated grain development. *OsERF115* encodes an AP2/ERF‐type transcriptional factor that is specifically expressed in young spikelets and developing caryopses. Overexpression of *OsERF115* significantly increases grain length, width, thickness and weight by promoting longitudinal elongation and transverse division of spikelet hull cells, as well as enhancing grain‐filling activity, whereas its knockout mutations lead to the opposite effects, suggesting that *OsERF115* positively regulates grain size and weight. *OsERF115* transcription is strongly induced by ethylene, and OsEIL1 directly binds to the promoter to activate its expression. OsERF115 acts as a transcriptional repressor to directly or indirectly modulate a set of grain‐size genes during spikelet growth and endosperm development. Importantly, haplotype analysis reveals that the SNP variations in the EIN3‐binding sites of *OsERF115* promoter are significantly associated with the *OsERF115* expression levels and grain weight, suggesting that natural variations in the *OsERF115* promoter contribute to grain‐size diversity. In addition, the *OsERF115* orthologues are identified only in grass species, implying a conserved and unique role in the grain development of cereal crops. Our results provide insights into the molecular mechanism of ethylene‐mediated grain‐size control and a potential strategy based on the OsEIL1‐OsERF115‐target gene regulatory module for genetic improvement of rice yield.

## Graphical abstract

The transcriptional induction of *OsERF115* by ethylene depends on OsEIL1 directly activating its expression. OsERF115 regulates the downstream genes to enhance cells proliferation and expansion, ultimately leading to an increase in grain size in rice.

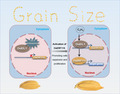

**Highlights**

- Enhancing of *OsERF115* expression significantly increases grain size by promoting expansion or proliferation of spikelet hull and endosperm cells.
- *OsERF115*can respond to ethylene signalling, and OsEIL1 directly binds to the EIN3‐binding site (EBS) motif on its promoter and activates its expression.
- As a transcriptional repressor, OsERF115 directly inhibits the expression of the grain size negative regulatory genes *OsGW2* and *OsGS6*.
- The −835 SNP is located at the EBS in *OsERF115* promoter and significantly associated with 1000‐grain weight.
- OsERF115 is a unique member of the ERF family in grass species.

## Introduction

Rice (*Oryza sativa* L.) is an important staple food crop in the world. Grain size is one of the essential determinants of rice yield. Therefore, understanding the mechanism of grain‐size formation is crucial for genetic improvement of rice yield potential (Li *et al*., 2018). Grain size is determined by its length, width and thickness. Grain length and width are determined by cell number and size in spikelet hulls, while grain thickness is mainly affected by grain‐filling activity (Bai *et al*., 2012). Phytohormones are important regulators of organ size in plants (Mizukami, 2001). Extensive studies have demonstrated that brassinosteroid (BR), auxin, cytokinin (CK) and gibberellin (GA) play important roles in controlling rice grain size by affecting cell proliferation and expansion in spikelet hulls and/or regulating endosperm development (Azizi *et al*., 2019; Li *et al*., 2019; Shi *et al*., 2020). Nevertheless, the knowledge about grain‐size control by ethylene is quite limited.

The gaseous hormone ethylene plays important roles in many aspects of plant growth and development, such as cell division, expansion and differentiation, seed germination, seedling emergence, root development, sex determination, fruit ripening, organ senescence, and responses to biotic and abiotic stresses (Zhao *et al*., 2021). Ethylene is synthesized from the methionine (Met) via a three‐step pathway, in which conversion of activated Met into the ethylene precursor 1‐aminocyclo‐propane‐1‐carboxylic acid (ACC) by ACC synthase (ACS) is the major rate‐limiting step (Ma *et al*., 2014). Ethylene is perceived by a family of receptors that negatively regulate ethylene responses. The signal is transmitted through a linear pathway, consisting of the negative regulator CONSTITUTIVE TRIPLERESPONSE 1 (CTR1), the positive regulator ETHYLENE INSENSITIVE 2 (EIN2), the master transcription factors EIN3 and EIN3‐LIKE1 (EIL1), and the ethylene response factors (ERFs) (Ma *et al*., 2014; Zhao *et al*., 2021). ERFs belong to a large gene family of plant‐specific transcription factors (TFs) containing a single AP2/ERF domain and the rice genome contains 139 ERF members (Nakano *et al*., 2006). ERFs are crucial downstream effectors of ethylene signalling pathway (Xie *et al*., 2019). In the presence of ethylene, EIN3/EILs activate *ERF* gene expression to initiate transcriptional cascades that ultimately activate or repress expression of various ethylene‐responsive genes.

The effect of ethylene on rice grain size has long been ambiguous (Yin *et al*., 2017), although several reports suggested a negative impact of ethylene on grain filling in inferior spikelets, based on chemical treatments with ethylene‐releasing agent ethephon or inhibitors (Naik and Mohapatra, 2000; Panda *et al*., 2016; Yang *et al*., 2006). The lack of ethylene‐related mutants has largely hampered the understanding of the exact roles of ethylene in rice growth and development (Ma *et al*., 2010). Recently, we have isolated a set of ethylene‐responsive mutants in rice (named *mao huzi*, *mhz*) (Ma *et al*., 2013). Studies on the *mhz* mutants and transgenic plants reveal a positive requirement for ethylene signalling in controlling grain size and weight. For instance, the loss of function of positive regulator genes in ethylene signalling pathway, such as *Osein2/mhz7*, *Oseil1/mhz6* or *mhz3* (an OsEIN2‐interacting protein), results in complete ethylene insensitivity and significant reduction in grain size and 1000‐grain weight, whereas overexpression of these genes substantially increases grain size (Ma *et al*., 2013, 2018; Yang *et al*., 2015). Consistently, RNAi knockdown of the ethylene receptor gene *OsETR2* enhances ethylene sensitivity and increases 1000‐grain weight, while the opposite effects are observed in *OsETR2*‐overexpressing plants (Wuriyanghan *et al*., 2009). Collectively, these findings provide convincing evidences for the positive roles of ethylene in controlling rice grain size. However, the downstream effectors and specific regulatory mechanism remain to be elucidated.

Here, we identify a seed‐specific TF OsERF115 as a key effector regulating rice grain size downstream of ethylene signalling pathway. *OsERF115* is transcriptionally induced by ethylene, and is directly activated by TF OsEIL1. We demonstrate that *OsERF115* positively regulates rice grain size by promoting longitudinal elongation and transverse division of spikelet hull cells and accelerating grain‐filling rate, thereby leading to an increase in rice grain yield. Furthermore, we find that OsERF115 acts as a transcriptional repressor to directly or indirectly regulate a set of grain size‐related genes. Our findings shed light on the molecular mechanism of ethylene controlling grain size, and the OsEIL1‐OsERF115‐grain‐size genes regulatory module provide a potential strategy for improving grain yield in rice.

## Results

### *OsERF115* is a grass‐specific gene that specifically expresses in rice spikelets and develops caryopses, and positively regulates grain development

To identify the potential effectors controlling rice grain size downstream of ethylene signalling pathway, we searched for seed‐specific *OsERF* members using the Rice Expression Profile (RiceXPro) Database (http://ricexpro.dna.affrc.go.jp/). Among the total 139 *OsERF* genes, *OsERF044* (LOC_Os08g45110) and *OsERF115* (LOC_Os08g41030) exhibited specific expression patterns in ovary, endosperm and/or embryo (Figure S1A). To test whether the two *ERFs* are ethylene‐responsive genes, we detected their expression levels in wild‐type (WT) rice plants and ethylene‐insensitive mutant *Osein2*. Reverse transcription quantitative PCR (RT‐qPCR) analysis showed that the expression of *OsERF115* was severely depressed in *Osein2* mutant compared with WT, whereas *OsERF044* transcript level was not affected by *Osein2* mutation (Figure S1B), indicating that *OsERF115* may act as a potential downstream effector of ethylene signalling pathway. Moreover, overexpression analysis showed that *OsERF115* could obviously increase grain size (Figure S2A,B), suggesting that *OsERF115* plays an important role in rice grain development.

**Figure 1:**
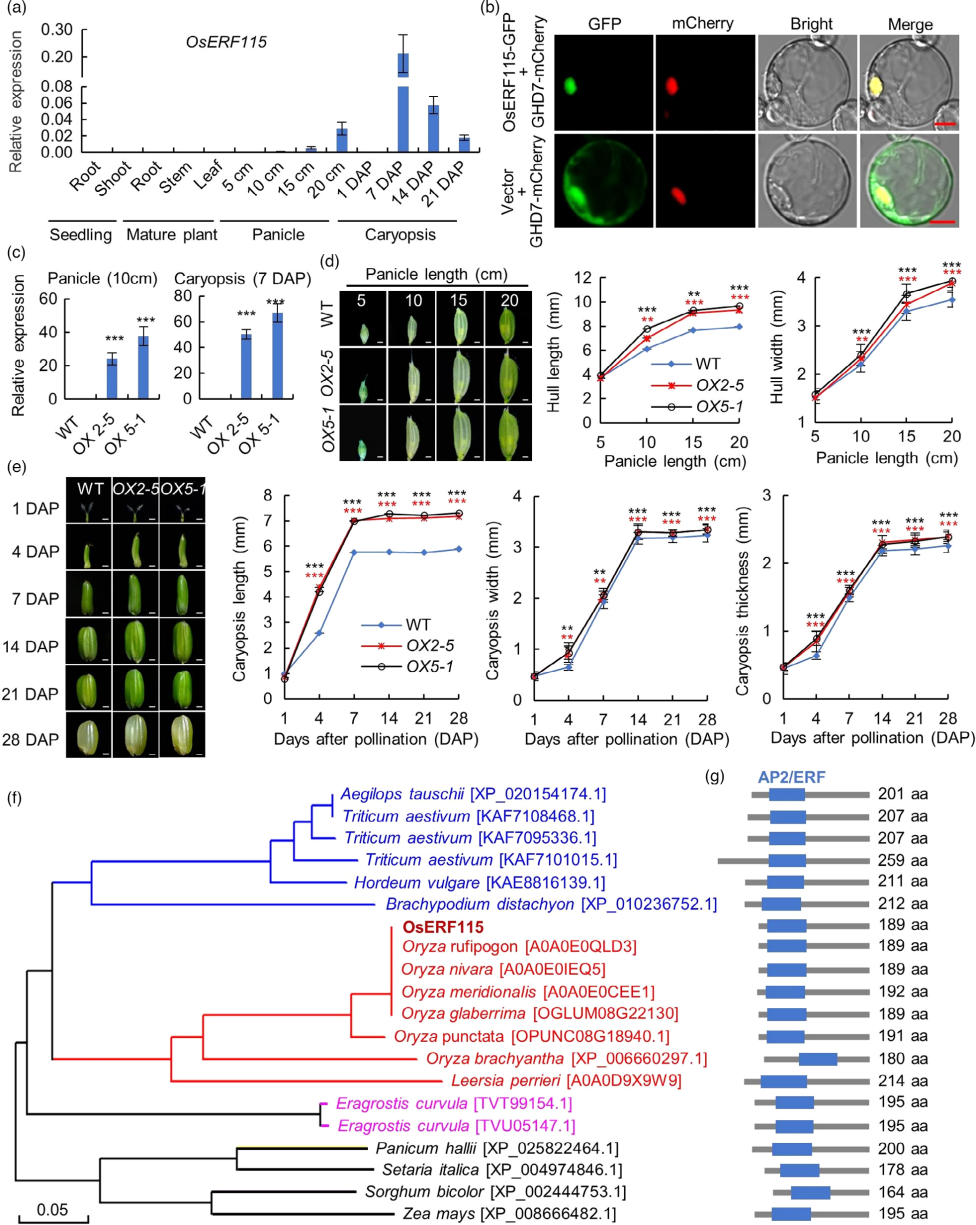
Characterization of *OsERF115* gene. (a) RT‐qPCR analysis of *OsERF115* expression in various organs of rice plants (*Japonica* variety Nipponbare). Data are means ± SD, *n* = 3. (b) Subcellular localization of OsERF115 in rice protoplasts. GHD7‐mCherry plasmid was used as a nuclear marker. Scale bars, 10 μm. (c) RT‐qPCR analysis of *OsERF115* expression in its two overexpressing (OX) lines. Data are means ± SD, *n* = 3. (d, e) Growth analysis of spikelet hulls (*d*) and developing caryopses (*e*) in WT and two *OsERF115*‐OX lines. Scale bars, 1 mm. Statistical data are means ± SD, *n* = 30. (f) Phylogenetic tree of OsERF115 and its orthologues in plant species. Phylogenetic construction was performed using DNAMAN v8 software (Lynnon Biosoft) with the default parameters. (g) Schematic diagram of the protein structures of OsERF115 and its orthologues in plant species corresponding to (*f*). The AP2/ERF domains are indicated as blue boxes, and the protein lengths are shown on the right. **P < 0.01, ***P < 0.001, Student’s *t*‐test.

To clarify the exact function of *OsERF115* in grain‐size control, we examined its spatiotemporal expression in various organs/tissues with different developmental stages using RT‐qPCR analysis. *OsERF115* transcripts were specifically detected in young panicles and developing caryopses (Figure 1a). The transcript levels in young panicles were quite low, but increased continuously along with the spikelet growth. *OsERF115* abundantly expressed in developing caryopses from 1 to 21 days after pollination (DAP) with the expression peak at 7 DAP stage. In addition, subcellular localization analysis showed that OsERF115 proteins are predominantly localized in the nucleus of rice protoplast cells, supporting its TF function (Figure 1b). These results suggest that OsERF115 likely functions as a transcriptional regulator in both spikelet growth and early seed development. To further elucidate *OsERF115* function, we generated overexpressing lines of *OsERF115* in the *japonica* variety Nipponbare and the transgenic plants showed significant increases in grain size (Figure S2A,B). The copy numbers of T‐DNA insertion in two representative overexpressing lines (*OX2‐5* and *OX5‐1*) were determined using droplet digital PCR (ddPCR). Among the T_2_ and T_3_ generation plants, both *OX2‐5* and *OX5‐1* harboured one copy of T‐DNA fragment; therefore, the two overexpressing lines were used for further studies (Figure S2C). RT‐qPCR analysis showed that the expression of *OsERF115* was severely increased in *OX2‐5* and *OX5‐1* compared with WT (Figure 1c). Furthermore, we examined the growth dynamics of spikelet hulls and developing caryopses in *OX2‐5* and *OX5‐1* (Figure 1d–e). Comparted with the WT, the spikelets of OX lines exhibited obvious increases in both hull length and hull width from 10 to 20 cm panicle stages (Figure 1d), and the caryopsis size significantly increased from 4 to 28 DAP stages in terms of length, width and thickness (Figure 1e). These observations indicate that *OsERF115* indeed plays significant roles in both spikelet hull growth and caryopsis development.

Interestingly, *OsERF115* has been clustered into a single‐member group XIV, which is distinct from the other OsERF members, and its orthologue was absent in *Arabidopsis* (Nakano *et al*., 2006). To understand the evolutionary context, we performed BLAST search of the OsERF115 protein sequence against multiple published databases (i.e. Gramene, Phytozome, NCBI and The Arabidopsis Information Resource). As a result, OsERF115 orthologues were identified only in grass species (Figure 1f,g), with 45.1%–100% protein sequence identity in *Oryzoideae*, 31.7%–42.9% identity in other grass species, and about 28% identity in nongrass species such as *Ananas comosus* and *Musa acuminata* (Table S1), suggesting that *OsERF115* may be a grass‐specific ERF family member.

### Overexpression of *OsERF115* significantly increases grain size and weight

**Figure 2:**
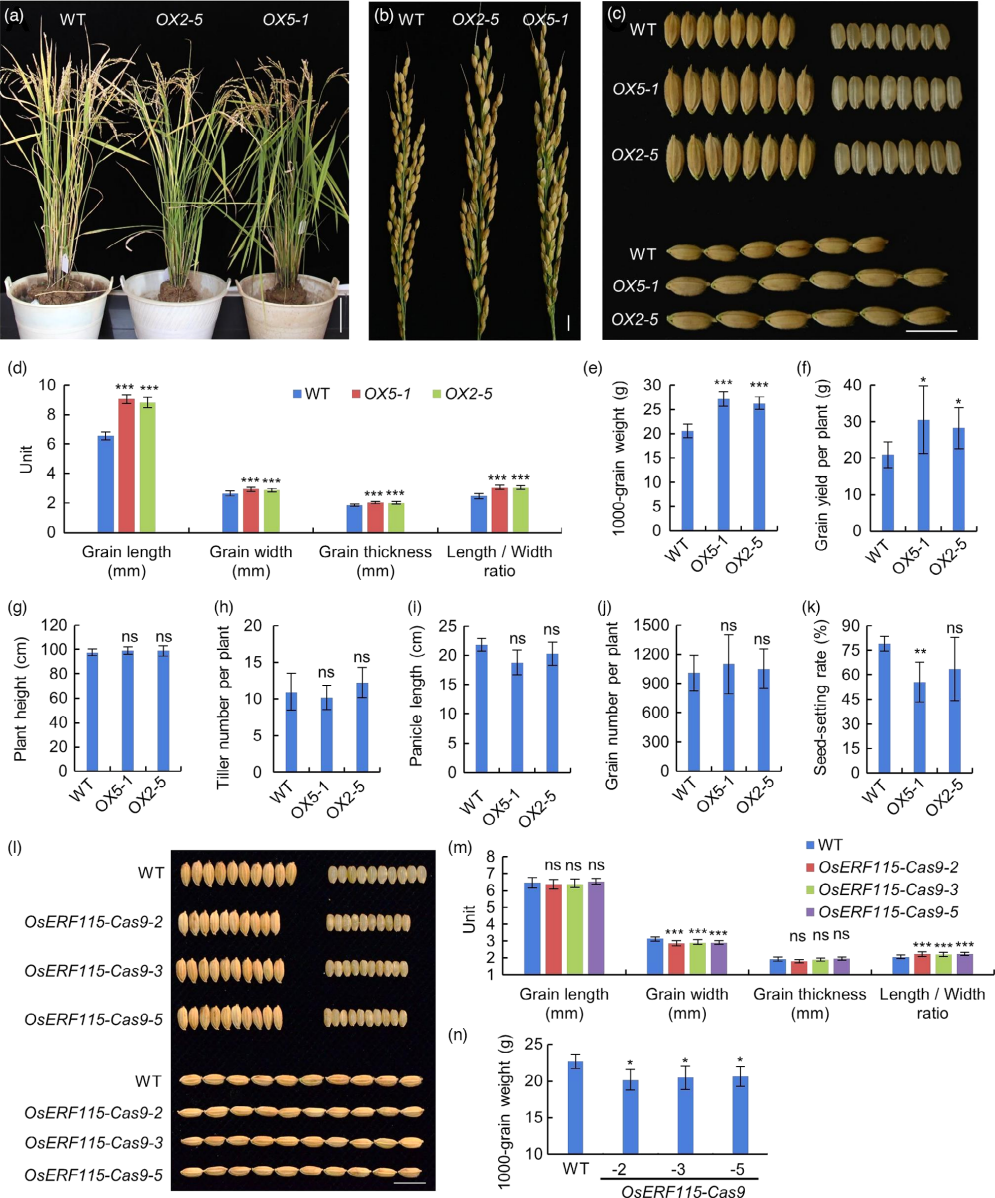
Phenotypic analysis of *OsERF115*‐overexpressing and CRISPR/Cas9 knockout rice lines. (a–c) Morphological comparison of whole plants (*a*), panicles (*b*) and grains (*c*) between WT and two *OsERF115*‐OX lines. Scale bars, 10 cm in ‘a’, 1 cm in ‘b’ and ‘c’. (d) Statistical analysis of grain size and shape in WT and two *OsERF115*‐OX lines. Data are means ± SD, *n* = 50. (E–K) Yield‐related traits in WT and two *OsERF115*‐OX lines. 1000‐grain weight (*e*), grain yield per plant (*f*), plant height (*g*), tiller number per plant (*h*), panicle length (*i*), grain number per plant (*j*) and seed‐setting rate (*k*). Data are means ± SD, *n* = 5‐8. (l) Grain morphology of WT and three *OsERF115* knockout lines generated by a CRISPR/Cas9 system. Scale bars, 1 cm. (m) Quantification of grain size and shape of WT and three *OsERF115* CRISPR/Cas9 knockout lines. Data are means ± SD, *n* = 50. (n) 1000‐grain weight of WT and three *OsERF115* knockout lines. Data are means ± SD, *n* = 4. *P < 0.05, **P < 0.01, ***P < 0.001, Student’s *t*‐test. ns, no significance.

We investigated the impact of *OsERF115* on yield‐related traits and found that overexpression of *OsERF115* had no significant influence on plant height, tiller number, panicle length and grain number (Figure 2a,b,g–j). By contrast, compared with WT, the mature grains of two *OsERF115*‐OX lines showed 35.0%–38.5% increase in length, 8.6%–11.1% increase in width, 9.2%–10.1% increase in thickness and 23.8%–24.2% increase in the ratio of length to width, thereby leading to 28.0%–32.0% increase in 1000‐grain weight (Figure 2c–e). Consistent with this predominantly positive effect of *OsERF115* on grain size and weight, the final grain yield per plant in *OX2‐5* and *OX5‐1* increased about 34.9% and 45.9%, respectively, although the seed‐setting rate in *OX5‐1* slightly decreased (Figure 2f,k). In addition to the results obtained above from the rice plants grown in Beijing, North China, the *OX2‐5* and *OX5‐1* lines grown in Hunan Province, South China, also exhibited a similar increase in grain size and led to 13.3%–18.2% increase in 1000‐grain weight (Figure S3), indicating that big‐grain phenotype of *OsERF115*‐OX lines is relatively stable under different environments. Moreover, overexpression of *OsERF115* in Kasalath, a small grain size *indica* variety, resulted in 21.3%–31.4% increase in grain length and 7.5%–17.3% increase in 1000‐grain weight, although no significant difference was observed in grain width and thickness between WT and the OX transgenic lines (Figure S4). These results together demonstrate that the up‐regulated expression of *OsERF115* can increase grain size and weight under different genetic backgrounds and diverse environmental conditions. To further confirm the gene function, we generated *OsERF115*‐knockout lines using a CRISPR/Cas9 genome‐editing system (Figure S5). By DNA sequencing, we identified the mutagenesis of *OsERF115* and obtained three loss‐of‐function mutants (*OsERF115*‐Cas9‐1, *OsERF115*‐Cas9‐2 and *OsERF115*‐Cas9‐3), which carried homozygous chromosomal deletion alleles due to 195‐bp, 146‐bp and 145‐bp deletion respectively (Figure S5A,B). RT‐qPCR analysis showed that the transcript levels of *OsERF115* significantly decreased in the three knockout lines compared with WT (Figure S5C). All the three independent knockout lines displayed significant reduction in grain width, resulting in slender grains and significant reduction of 1000‐grain weight (Figure 2l–m). This result further proved the positive impact of *OsERF115* on regulation of grain size and weight.

### *OsERF115* promotes longitudinal elongation and transverse division of spikelet hull cells and enhances grain‐filling activity by regulating a set of grain‐size genes

**Figure 3:**
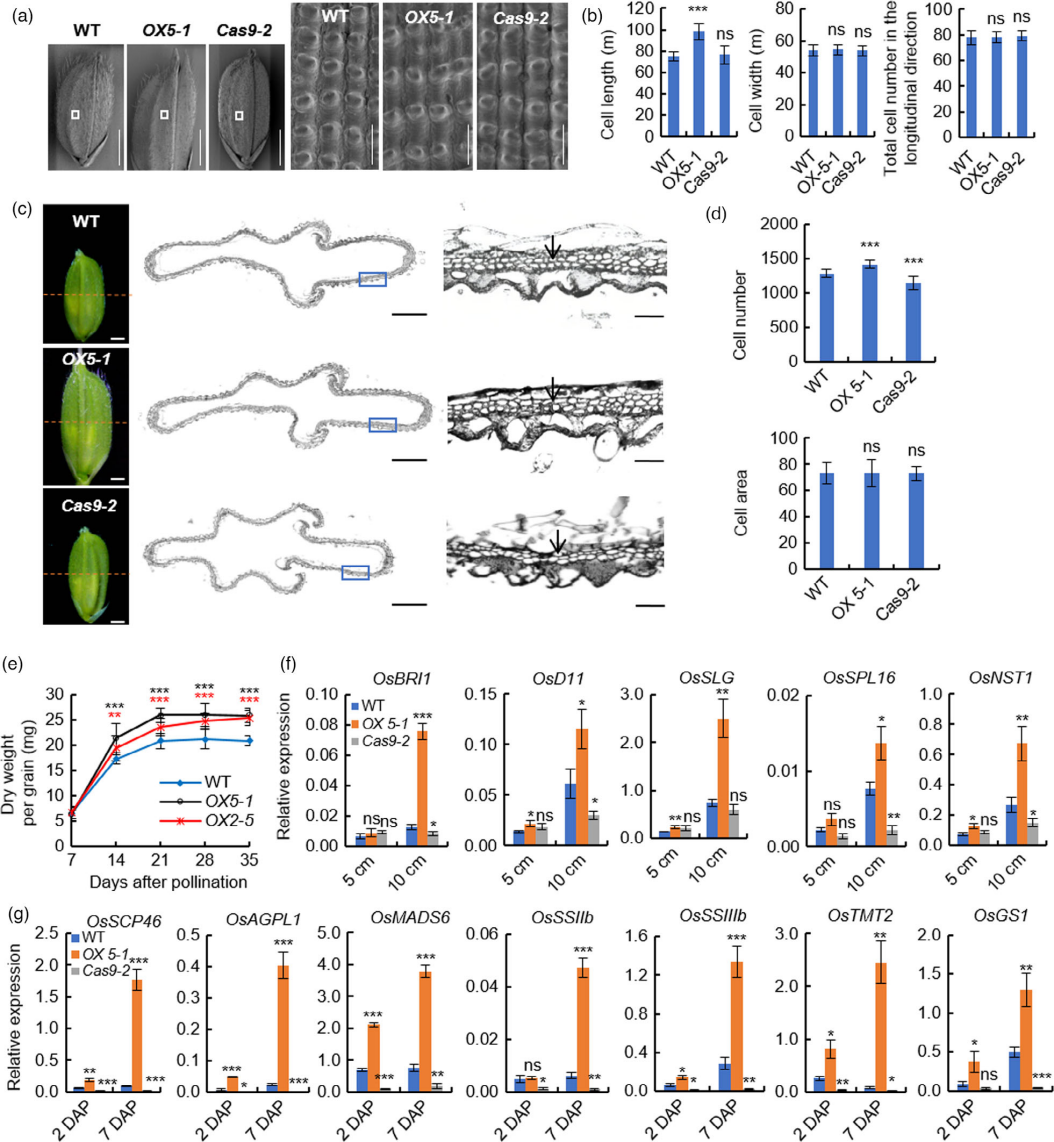
*OsERF115* promotes longitudinal elongation and transverse division of spikelet hull cells and enhances grain‐filling activity. (a) Scanning electron microscopy (SEM) observation of the glume outer surfaces of WT, *OX5‐1* and *Cas9‐2* lines mature grains. Scale bar, 2 mm for whole grains and 100 μm for outer glume. (b) Statistical analysis of cell length, width and number in the longitudinal direction in (*a*). (c) Cross‐sections of spikelet hulls of WT, *OX5‐1* and *Cas9‐2* lines. Scale bars, 1 mm for whole spikelets (left), 500 μm for cross‐sections (middle) and 100 μm for magnified views (right). Arrows indicate the outer parenchyma layer corresponding to the counted cells. (d) Quantification of cell number and area in the outer parenchyma layer of spikelet hulls. (e) Grain‐filling rate in WT and two *OsERF115‐*OX lines. (f) Expression analysis of grain length‐ and width‐controlling genes in young panicles of WT, *OX5‐1* and *Cas9‐2* lines by using RT‐qPCR. (g) Expression analysis of grain filling‐related genes in developing caryopses of WT, *OX5‐1* and *Cas9‐2* lines by using RT‐qPCR. Data are means ± SD, *n* = 10 (*b* and *d*), 20 (*e*), and 3 (*f* and *g*) respectively. *P < 0.05, **P < 0.01, ***P < 0.001, Student’s *t* test. ns, no significance.

Scanning electron microscopy (SEM) observation of the outer epidermis of mature grains showed that cell length in *OX5‐1* increased significantly, but no obvious difference in *Cas9‐2* line as compared with that of WT. In addition, no differences were observed in the cell width and longitudinal cell number in both *OX5‐1* and *Cas9‐2* lines (Figure 3a,b). To confirm the SEM results of mature grains, we performed longitudinal‐section analysis of the spikelet hulls. Similarly, increased epidermal cell length was observed in *OX5‐1*, but no change in *Cas9‐2*, and no differences in epidermal cell number were detected in both *OX5‐1* and *Cas9‐2* (Figure S6A,B). All these cytological analyses suggest that the increased grain length of *OX5‐1* mainly results from the enhanced cell elongation but not cell division in the longitudinal direction of spikelet hulls, and loss of function of *OsERF115* does not affect the cell development of spikelet hulls in the longitudinal direction. Next, cross‐section observation of spikelet hulls just before flowering found that the cell number in the outer parenchyma layer increased in *OX5‐1* and decreased in *Cas9‐2* significantly, but no obvious difference in cell size as compared with that of WT. These data indicate that the cell number in transverse direction of spikelet hulls rather than the cell area, contributes to the altered grain width of *OX5‐1* and *Cas9‐2* (Figure 3c,d). To understand how *OsERF115* influences grain weight, we compared the cell number and size within the developing endosperms. Cross‐section observation of 7 DAP caryopses found that the cell number of aleurone layer significantly increased in *OX5‐1* and substantially decreased in *Cas9‐2*, although there was no obvious difference in cell size as compared with that of WT (Figure S6C,D). Moreover, time course analysis of grain dry weight showed that the grain‐filling rates in *OX2‐5 and OX5‐1* plants were significantly higher than that of WT from 14 to 35 DAP, suggesting that the increased grain thickness and weight of OX2‐5 and OX5‐1 lines are mainly caused by the accelerated grain‐filling rates (Figure 3e). Together, *OsERF115* increases rice grain length and width by promoting longitudinal elongation and transverse division of spikelet hull cells and improves grain thickness and weight via enhancing division of endosperm cells and grain‐filling activity.

To further elucidate the molecular bases underlying the histological and physiological observations, we performed RNA‐seq analysis using 5‐ and 10‐cm panicles as well as 2 DAP and 7 DAP caryopses of WT and *OX5‐1* line. Based on the transcriptome data, we identified a set of grain‐size genes differently expressed between WT and *OX5‐1* samples (Figure S7). RT‐qPCR analysis was performed to further confirm the expression profiles. In young panicles, five genes that positively regulate spikelet hull growth were substantially up‐regulated in *OX5‐1* comparted with WT, and down‐regulated in *Cas9‐2* except *OsSLG* (Figure 3f). Among them, the BR receptor gene *OsBRI1/D61*, BR biosynthesis gene *OsD11/CPB1* and BR homeostasis‐related gene *OsSLG* have been proved to increase grain length by influencing cell elongation (Feng *et al*., 2016; Morinaka *et al*., 2006; Wu *et al*., 2016); the SBP family TF *OsSPL16/GW8* can increase grain width by promoting cell proliferation (Wang *et al*., 2012); and *OsNST1* controls grain size by modulating cell wall biosynthesis (Zhang *et al*., 2011). Hence, it is reasonable to conclude that *OsERF115* positively regulates grain length and width through activating these genes. In developing caryopses, seven genes that act as positive regulators of grain filling were significantly up‐regulated in *OX5‐1* and down‐regulated in *Cas9‐2* comparted with WT, including *OsSCP46*, *OsAGPL1*, *OsMADS6*, *OsSSIIb*, *OsSSIIIb*, *OsTMT2* and *OsGS1* (Figure 3g). These genes are reported to be involved in endosperm development, starch synthesis, sugar transport or carbon–nitrogen metabolic processes (Cho *et al*., 2010; Li *et al*., 2016). Accordingly, it appears that *OsERF115* increases grain thickness and weight by up‐regulating expressions of these grain‐filling genes.

### Ethylene induces *OsERF115* gene expression through OsEIL1‐mediated direct transcriptional activation

**Figure 4:**
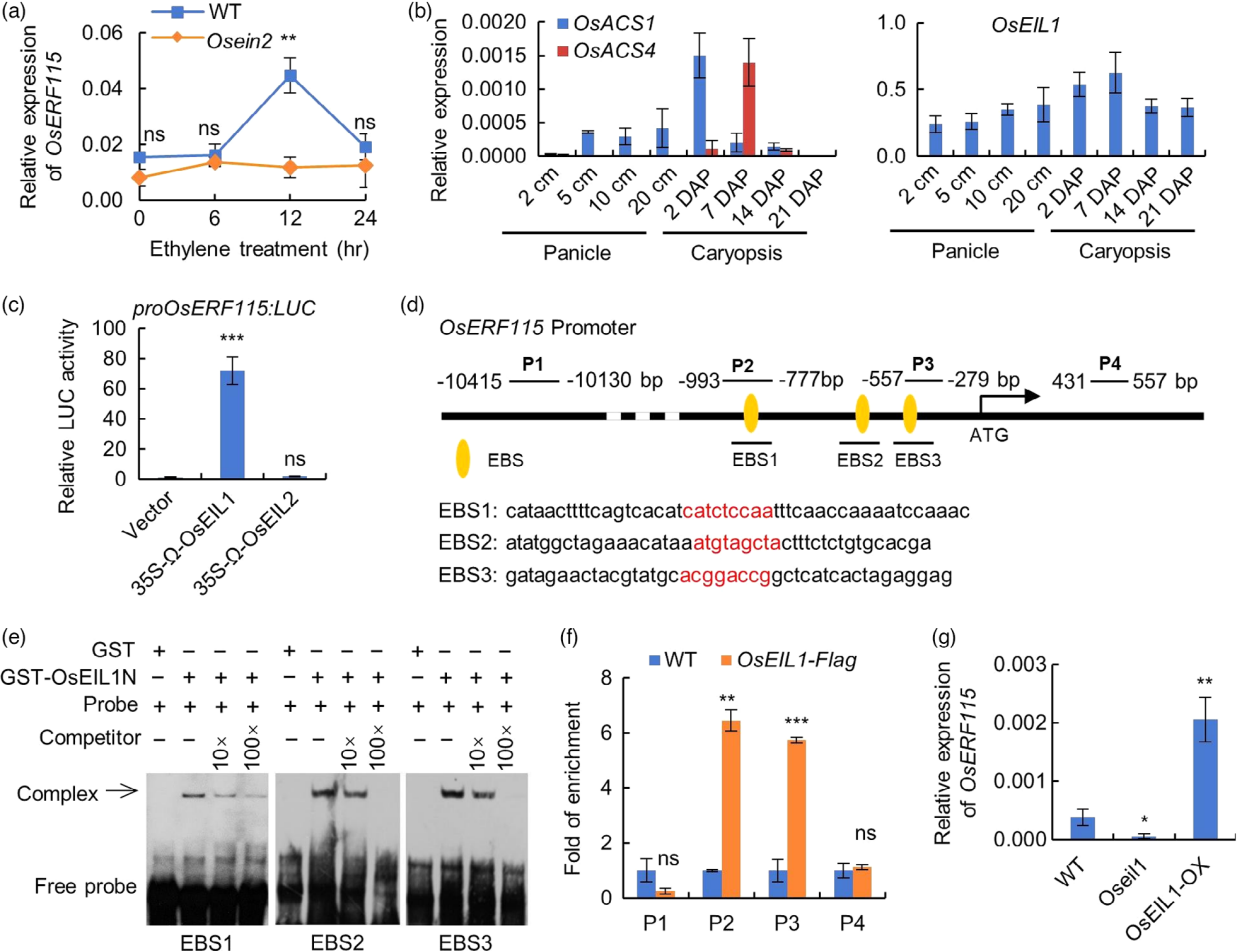
Ethylene induces *OsERF115* expression through OsEIL1‐mediated direct transcriptional activation. (a) Induction of *OsERF115* expression by ethylene treatment (100 ppm) in 10 cm panicles of WT and *Osein2* mutant as revealed by RT‐qPCR analysis. (b) Expression levels of *OsACS1*, *OsACS4* and *OsEIL1* genes in WT young panicles and developing caryopses analysed by RT‐qPCR. (c) Dual‐luciferase assay of *OsERF115* promoter activity activated by OsEIL1 or OsEIL2 in rice protoplasts. (d) Four probe positions on *OsERF115* promoter and coding region. Probe 2 (P2) and probe 3 (P3) contain the EIN3 binding site (EBS). The EBS sequences in EBS1, EBS2 and EBS3 are shown in red letters. (e) EMSA assay of OsEIL1 binding to the *OsERF115* promoter region containing the EBS. (f) The enrichments of *OsERF115* promoter analysed by ChIP‐qPCR using the *35S:OsEIL1‐Flag* transgenic rice plants. WT plants were used as a negative control. (g) Expression levels of *OsERF115* in 10‐cm panicles of WT, *Oseil1* mutant and *OsEIL1‐*OX plants analysed by RT‐qPCR. Data are means ± SD, *n* = 3 (*a–c*, *f* and *g*). *P < 0.05, **P < 0.01, ***P < 0.001, Student’s *t* test. ns, no significance.

To clarify whether *OsERF115* is an ethylene downstream effector, we examined the gene expression in response to ethylene treatment. RT‐qPCR analysis showed that *OsERF115* transcripts were obviously induced in WT young panicles by treatment with exogenous ethylene, whereas the induction was completely blocked in *Osein2* mutant, demonstrating that *OsERF115* is an ethylene‐responsive gene (Figure 4a). Gene expression analysis of WT young panicles and developing caryopses found that the expression patterns of ethylene biosynthesis genes such as *OsACS1* and *OsACS4* and the master TF gene *OsEIL1* were similar to that of *OsERF115*, implying that activation of *OsERF115* in rice plants possibly depends on endogenous ethylene signals (Figure 4b). Next, to determine the upstream regulator of *OsERF115*, we performed a dual‐luciferase transactivation assay in rice protoplasts using OsEIL1 and OsEIL2 as effectors, as only the two members among the six OsEILs have been shown to regulate ethylene response in rice (Yang *et al*., 2015). The *OsERF115* promoter was significantly activated by OsEIL1, but not by OsEIL2, suggesting that OsEIL1 can serve as the upstream activator of *OsERF115* (Figure 4c). Sequence analysis of the *OsERF115* promoter revealed three putative EIN3‐binding sites (EBSs) (Figure 4d). Thus, we investigated the possibility of OsEIL1 binding to the EBS motifs using an electrophoretic mobility shift assay (EMSA). As a result, the three fragments harbouring EBS1, EBS2 and EBS3 were specifically bound by OsEIL1 protein, indicating that OsEIL1 can directly bind to the *OsERF115* promoter *in vitro* (Figure 4e). Moreover, chromatin immunoprecipitation quantitative PCR (ChIP‐qPCR) analysis using the *35S:OsEIL1‐Flag* transgenic rice plants showed that OsEIL1 bound to the *OsERF115* promoter *in vivo* via specific association with the EBS motifs (Figure 4f). Furthermore, RT‐qPCR analysis showed that *OsERF115* expression was significantly inhibited in *Oseil1* mutant, but was greatly up‐regulated in *OsEIL1*‐OX rice plants (Figure 4g). Collectively, the data convincingly demonstrate that *OsERF115* is a vital downstream effector of ethylene signalling pathway and OsEIL1 directly activates its expression.

### *OsERF115* functions as a transcriptional repressor and directly represses *OsGW2* and *OsGS6* gene expression

**Figure 5:**
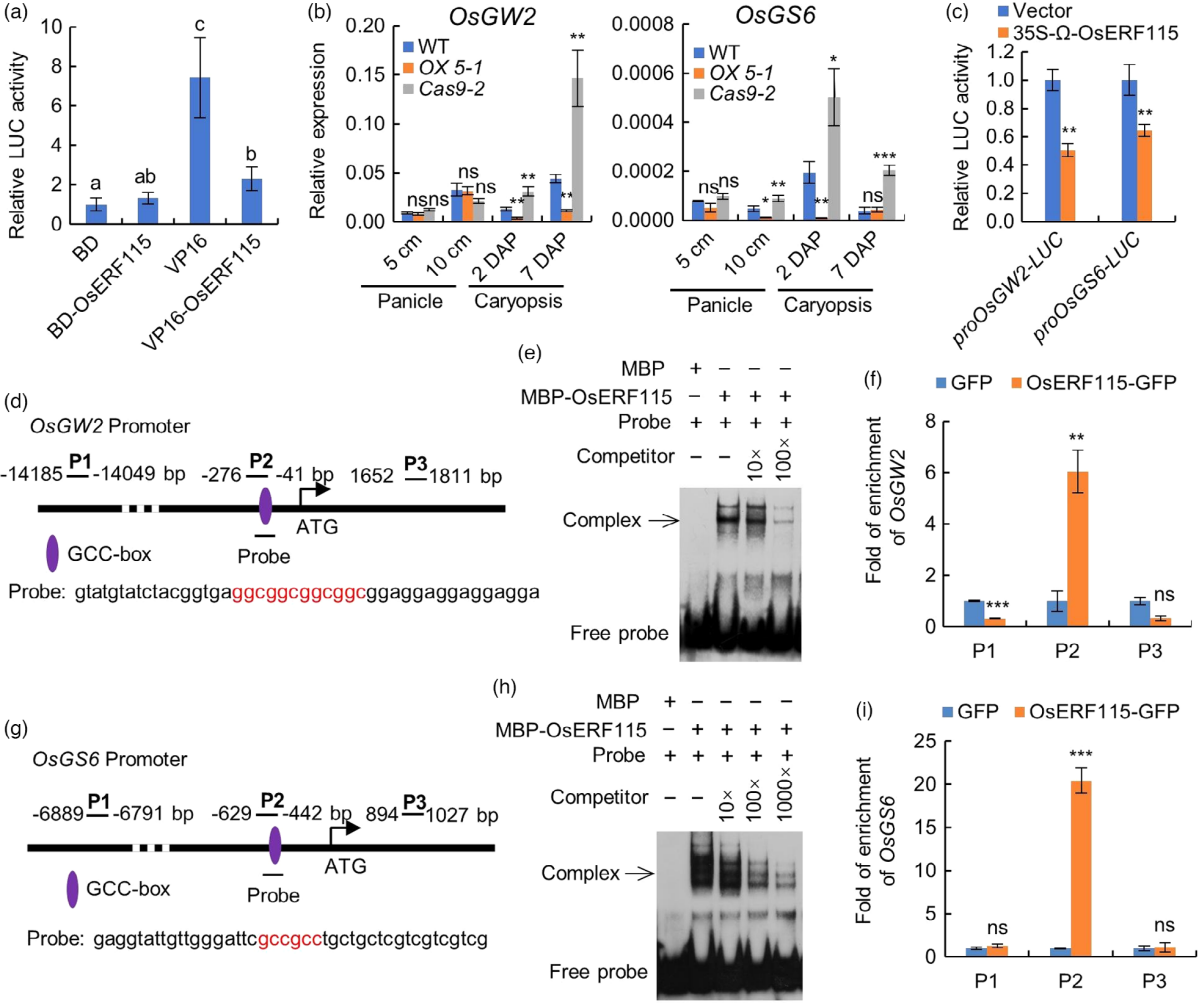
*OsERF115* functions as a transcriptional repressor and directly represses *OsGW2* and *OsGS6* gene expression. (a) Transactivation assay of *OsERF115* in rice protoplasts using dual‐luciferase reporter system. Different letters indicate significant differences (*P* < 0.01, LSD test) in the multiple comparison. (b) Expression levels of *OsGW2* and *OsGS6* in panicles and caryopses of WT, *OX5‐1* and *Cas9‐2* lines analysed by RT‐qPCR. (c) Dual‐luciferase assay of promoter activities of *OsGW2* and *OsGS6* inhibited by TF OsERF115 in rice protoplasts. (d–i) Direct binding of OsERF115 to the promoters of *OsGW2* and *OsGS6* both *in vivo* and *in vitro*, as revealed by EMSA assay (*e* and *h*) and ChIP‐qPCR analysis using *35S:OsERF115‐GFP* transgenic rice plants (*f* and *i*). The *35S:GFP* transgenic rice plants were used as a negative control for ChIP‐qPCR analysis. The GCC‐box sequences are shown in red letters in (*d*) and (*g*). Data are means ± SD, *n* = 3 (*a–c*, *f* and *i*). *P < 0.05, **P < 0.01, ***P < 0.001, Student’s *t* test. ns, no significance.

To determine the transcriptional activity of OsERF115 protein, we performed dual‐luciferase transactivation assay using BD‐OsERF115 and VP16‐OsERF115 as effectors. Compared with the controls, OsERF115 significantly repressed the transcriptional activation activity of VP16, indicating that OsERF115 functions as a transcriptional repressor (Figure 5a). Next, we identified the downstream genes and processes potentially regulated by *OsERF115* based on the DEGs between WT and *OX5‐1* (fold change ≥2, FDR <0.005). A total of 185 and 604 DEGs were detected in 5‐cm and 10‐cm panicles, respectively, and 2392 and 2774 DEGs were detected in 2 DAP and 7 DAP caryopses respectively (Figure S7A and Table S2–S5). Gene ontology (GO) analysis revealed significant enrichment of ‘regulation of gene expression’ (GO: 0010468), which is consistent with the TF function of OsERF115 (Figure S7B). GO terms in 10‐cm panicles were enriched in four biological processes including translation, gene expression, protein metabolic process and structural constituent of ribosome, indicative of active cell growth and division. GO terms in 2 DAP and 7 DAP caryopses were mainly enriched for categories related to endosperm development, such as cell differentiation, carbohydrate biosynthetic and metabolic processes, and polysaccharide biosynthetic and metabolic processes (Figure S7B). Next, to identify the direct target genes of *OsERF115*, we searched for negative regulators of grain size in down‐regulated DEGs, based on the consideration that *OsERF115* acts as a transcriptional repressor, but positively regulates grain size. As a result, a total of four genes were identified, including *OspPLAIIα*, *OsGW2*, *OsPUP7* and *OsGS6* (Figure S7C). RT‐qPCR analysis showed that *OsGW2*, *OsGS6* and *OspPLAIIα* were significantly reduced in expression levels in 2 DAP or 7 DAP caryopses of *OX5‐1* when compared with that of WT (Figure 5b and Figure S8A). Furthermore, dual‐luciferase transactivation assay showed that OsERF115 could significantly repress the promoter activities of *OsGW2* and *OsGS6*, but had no effects on the promoters of the negative regulators *OspPLAIIα* and *OsPUP7* and the positive regulators of spikelet hull growth and grain filling (Figure 5c and Figure S8B,C,D). The results suggest that only *OsGW2* and *OsGS6* may be direct targets of OsERF115. Consistently, both promoters of *OsGW2* and *OsGS6* contained GCC box motifs, the *cis*‐acting element of AP2/ERF TFs (Figure 5d,g). EMSA experiments showed that OsERF115 protein specifically bound to the GCC box‐containing promoter regions, indicating that OsERF115 can directly bind to *OsGW2* and *OsGS6* promoters *in vitro* (Figure 5e,h). To further verify these protein–DNA interactions *in vivo* by ChIP‐qPCR analysis, we generated *35S:OsERF115‐GFP* transgenic rice plants, which displayed increased grain size and 1000‐grain weight, indicating that the *OsERF115‐GFP* fusion gene is functional (Figure S9). Compared with the control probes (P1 and P3), both P2 fragments containing GCC‐box were significantly enriched by ChIP assays using anti‐GFP antibody, indicating that OsERF115 can bind to *OsGW2* and *OsGS6* promoters *in vivo* (Figure 5f,i). This result further proved that *OsGW2* and *OsGS6* are the direct targets of TF OsERF115. Given that *OsGW2* represses endosperm cell expansion and *OsGS6* inhibits cell proliferation (Song *et al*., 2007; Sun *et al*., 2013), we propose that *OsERF115* promotes early endosperm development by directly repressing the expression of the negative regulators such as *OsGW2* and *OsGS6* and indirectly activating the positive modulators.

Considering the OsEIL1 act as positive regulators of grain size and weight (Yang *et al*., 2015), we examined the expression of *OsERF115* downstream genes in the *OEIL1‐OX* and *Oseil1* mutant by RT‐qPCR analysis. In young panicles, five genes that act as positive regulators of spikelet hull growth were substantially up‐regulated in *OEIL1‐OX* and down‐regulated in *Oseil1* comparted with WT (Figure S10A). In developing caryopses, seven genes that positively regulate grain filling were significantly up‐regulated in *OEIL1‐OX* and down‐regulated in *Oseil1* comparted with WT (Figure S10B). By contrast, the direct target genes of OsERF115 *OsGW2* and *OsGS6*, the negative regulators of grain filling, were significantly reduced in expression levels in 2 DAP or 7 DAP caryopses of *OEIL1‐OX* and dramatically up‐regulated in *Oseil1* compared with that of WT (Figure S10C). Collectively, *OsEIL1* displayed similar regulatory effects to that of *OsERF115* on its downstream genes. This result, combined with the OsEIL1‐mediated direct transcriptional activation of *OsERF115*, suggests that *OsEIL1* and *OsERF115* likely function in the same pathway and form a regulatory module to control rice grain development.

### SNPs in the EBS motif of *OsERF115* promoter are closely associated with the expression level of *OsERF115* and rice grain weight

**Figure 6:**
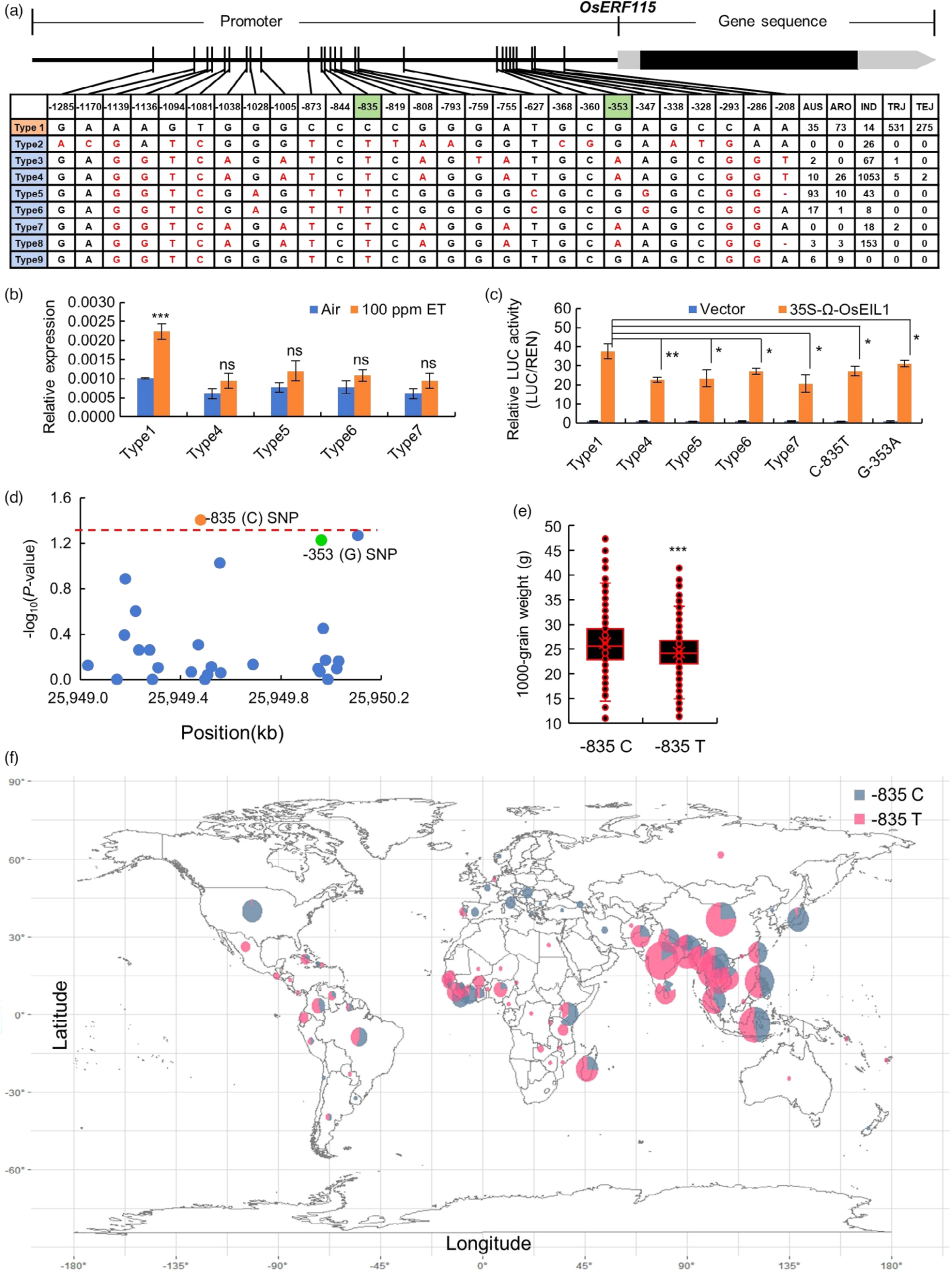
Polymorphisms in *OsERF115* promoter are associated with gene expression and grain weight in rice. (a) Haplotype analysis of the *OsERF115* gene region in 2486 rice cultivars. AUS *aus* population, ARO *aromatic* population, IND *indica* population, TRJ tropical *japonica* population, TEJ temperate *japonica* population. (b) Expression levels of *OsERF115* in the five haplotypes with ethylene treatment analysed by RT‐qPCR. Data are means ± SD, *n* = 3. ***P < 0.001, Student’s *t* test. ns, not significant. (c) Promoter activity analysis of the *OsERF115* promoters with different variations and site‐directed mutations at the two SNPs activated by OsEIL1 in *Oseil1* protoplasts. Data are means ± SD, *n* = 3. The asterisks indicate significant differences compared with Type 1 promoter activated by OsEIL1. *P < 0.05, **P < 0.01, Student’s *t* test. (d) Association testing between 1000‐grain weight and SNPs in the promoter. The dots marked in orange or green represent the SNPs located at EBSs. (e) Comparisons of 1000‐grain weight sorted by the −835 position alleles. *n* = 542 (−835C), 1363 (−835T). ***P < 0.001, Student’s *t*‐test. (f) Geographic origin of rice accessions containing the −835 position alleles.

To access the contribution of natural variations in *OsERF115* to rice grain size and weight, nucleotide polymorphisms in the promoter and coding region of *OsERF115* were investigated among 3010 rice accessions from the 3K Rice Genomes Project (Wang *et al*., 2018). After eliminating the heterozygotes and the haplotypes with less than five varieties, a total of 2486 accessions were retained and used for further analysis, in all of which no nucleotide difference was detected in the coding region, suggesting that the OsERF115 coding sequences are completely conserved in cultivated rice. Instead, 27 single‐nucleotide polymorphisms (SNPs) were detected in the promoter region (1.5 kb before ATG), and were divided into 9 haplotypes assigned to Types 1 to 9 (Figure 6a); 98.8% (806/816 accessions) *japonica* rice contained the Type1 haplotype that is consistent with the Nipponbare IRGSP 1.0 reference genome sequence, and 76.2% (1053 accessions) *indica* varieties contained the Type 4. Type1 and Type 4 haplotypes were distributed in 81.4% of the accessions. Other haplotypes were detected in one to three subpopulations other than *japonica*. To test the effects of these mutations on promoter activities, we sequenced the 1.5‐kb region of *OsERF115* promoters in 11 rice cultivars and found that they were classified as Types 4–7. We measured the response of *OsERF115* expression to ethylene treatment in five rice cultivars containing the Type 1 and Type 4–7 haplotypes, respectively. The result revealed that *OsERF115* transcripts were obviously induced by treatment with exogenous ethylene in cultivars possessing Type 1, whereas the induction was significantly impaired in other types, suggesting that the polymorphisms in *OsERF115* promoter may confer *OsERF115* transcriptional response to ethylene (Figure 6b). Next, we examined the promoter activities of these haplotypes using dual‐luciferase transactivation assay in WT or *Oseil1* protoplast cells and found that the promoter activity of Type1 was significantly higher than those of the other haplotypes either without or with OsEIL1 as an effector (Figure 6c and Figure S11). Remarkably, the −353, and −835 SNPs were located at the two EBSs (EBS2 and EBS1) which have been shown to be bound by OsEIL1 (Figure 4d,e) and Type1 harboured the intact EBSs (Figure 6a). To determine the effects of the two SNPs in EBSs, we conducted transient expression assays with the two site‐directed mutation promoter fragments of *OsERF115* in WT and *Oseil1* mutant protoplasts respectively. Compared with the Type1 promoter, the relative activities of mutated promoters (G‐353A and C‐835T) were significantly decreased and were comparable to the other haplotypes lacking at least one of the EBSs (Figure 6c and Figure S11). These results suggest that the SNPs that destroy the EBSs are mainly responsible for the promoter activity of *OsERF115* when compared with the other SNPs. Finally, to determine which variation in promoter region is related to grain size and weight, an association test between 1000‐grain weight and sequence variations was performed. As a result, we found strong signals at SNP site −835 in the promoter region (Figure 6d). We further compared grain weight sorted by the −835 position alleles among the rice cultivars and found the 1000‐grain weight was higher in rice cultivars with C allele (the intact EBS) than that with T allele (Figure 6e). Geographical distribution analysis showed that the rice accessions with −835C allele represented by japonica varieties increased with increasing latitude, whereas the −835T allele accessions were mainly located in low‐latitude regions (Figure 6f). This suggests that Type1 have a regional differentiation with other haplotypes. Taken together, the natural variations of EBSs in *OsERF115* promoter are responsible for rice grain size and weight likely by affecting *OsERF115* expression in response to endogenous ethylene.

## Discussion

**Figure 7:**
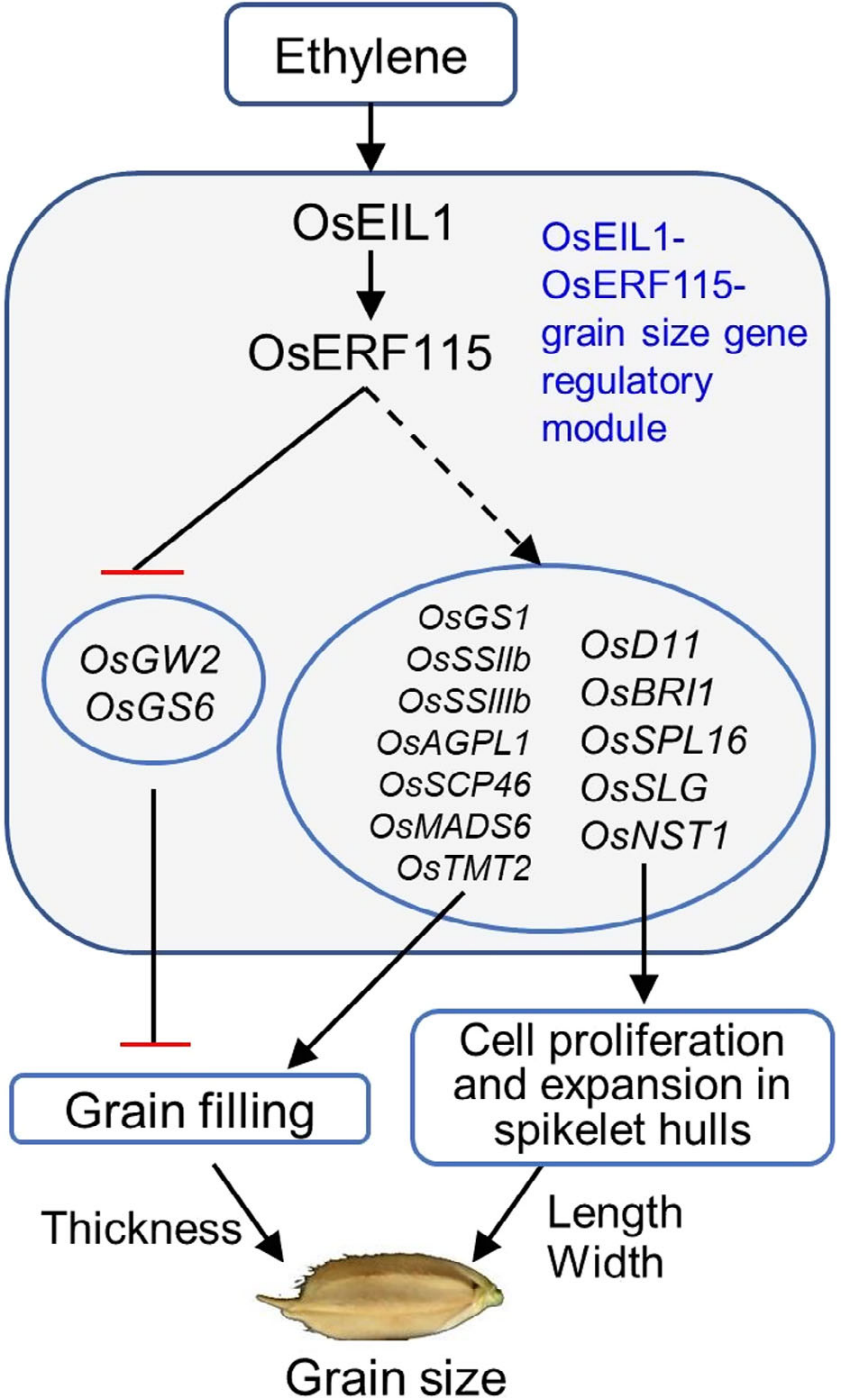
The proposed model for *OsERF115* mediating ethylene signalling to regulate grain size in rice. The transcriptional induction of *OsERF115* by ethylene depends on OsEIL1 directly activating its expression. OsERF115 indirectly promotes the positive regulatory genes of spikelet hull growth and endosperm development and directly represses *OsGW2* and *OsGS6* gene expression, thereby promoting grain filling and cell proliferation and expansion of spikelet hulls, ultimately leading to an increase in grain size.

In this study, multiple lines of evidence support that *OsERF115* is a key downstream effector of ethylene signalling pathway in rice grain‐size control. First, *OsERF115* is specifically expressed in rice young panicles and developing caryopses, where it is co‐expressed with ethylene biosynthesis and signalling genes. Second, *OsERF115* expression is strongly induced by ethylene treatment, and the master TF OsEIL1 directly activates its transcription. Third, overexpression of *OsERF115* increases grain size and weight, whereas the knockout mutations lead to the opposite effects, which is consistent with the promotive role of ethylene in grain‐size control reported previously (Ma *et al*., 2013, 2018; Wuriyanghan *et al*., 2009; Yang *et al*., 2015). Fourth, *OsERF115* increases grain length and width by promoting longitudinal elongation and transverse division of spikelet hull cells, respectively, and increases grain thickness and weight by enhancing division of endosperm cells and enhancing grain‐filling activity, which provides the cytological and physiological bases for *OsERF115*‐dependent grain‐size amplification. Fifth, *OsERF115* acts as a transcriptional repressor to directly or indirectly modulate a set of grain‐size genes during spikelet growth and endosperm development, illustrating the molecular basis of *OsERF115*‐meidated transcriptional regulation. Our results establish an OsEIL1‐OsERF115‐grain‐size gene regulatory module controlling grain size and weight in rice (Figure 7). Namely, ethylene induces *OsERF115* gene expression through direct transcriptional activation by the master TF OsEIL1. OsERF115 directly represses the expression of negative regulators such as *OsGW2* and *OsGS6* in grain filling and indirectly induces the positive regulatory genes of spikelet hull growth and endosperm development through some unknown factors, thereby positively modulating rice grain size and weight. However, it may also be possible that some co‐factors are required for OsERF115 to directly activate the positive regulatory genes. To clarify this, IP‐MS analysis is needed to identify the potential interacting proteins of OsERF115. Notably, the *OsERF115*‐Cas9 loss‐of‐function mutants only have altered grain width, while the OX lines have altered grain length, width and thickness. This may be caused by functional redundancy, as it has been reported that both OsERF114 and OsERF115 could interact with OsNF‐YB9 and OsNF‐YB7 to assemble a transcription factor complex to regulate endosperm development (E *et al*., 2018). However, we cannot rule out that possibility of artefacts conferred by ectopic expression of *OsERF115*.

So far, several *OsERF* genes have been shown to be involved in grain‐size control. *SERF1*/*OsERF131* (LOC_Os05g34730) acts as a transcriptional repressor and negatively regulates grain filling, but it is not an ethylene downstream effector (Schmidt *et al*., 2014). *FZP*/*OsERF078* (LOC_Os07g47330) functions as a transcriptional activator and positively influences grain size, whereas its ethylene responsiveness was not determined (Ren *et al*., 2018). *OsLG3*/*OsERF062* (LOC_Os03g08470) acts as a transcriptional activator contributing to grain length (Yu *et al*., 2017). *OsLG3* exhibits ethylene‐induced expression related to drought tolerance in rice seedlings, while its functional involvement in ethylene‐mediated grain‐size control is uncertain (Xiong *et al*., 2018). Notably, the expression levels of the three *OsERF* genes are not affected by *OsERF115* according to our RNA‐seq data (PRJNA701677), implying that these genes may function in different pathways with OsERF115 or may act downstream of it. OsERF115 has been identified as an interaction partner of OsNF‐YB1 protein that plays a regulatory role in endosperm development and grain filling (Xu *et al*., 2016). Nevertheless, little is known about the exact function of *OsERF115* as well as its relationship with the ethylene signalling pathway. In this study, the cytological, physiological and molecular biology analyses as well as the association test of 2486 rice accessions convincingly demonstrate that *OsERF115* regulates rice grain size and weight downstream of the ethylene signalling pathway. Our work establishes a direct link between ethylene signalling and grain‐size control via the OsEIL1‐OsERF115‐gran size gene regulatory molecule.

Generally, ethylene negatively affects organ size by restricting cell expansion/elongation (Mizukami, 2001). For instance, the *Arabidopsis* constitutive ethylene‐response mutant *ctr1* has smaller organs such as compact rosette leaves and inflorescences, short hypocotyls and roots and reduced seed mass, due to reduction in cell size (Ecker, 1995; Meng *et al*., 2018). Conversely, the ethylene‐insensitive mutant *ein2* plants bear larger rosette leaves, floral organs and seeds with enlarged cells relative to the WT plants (Feng *et al*., 2015; Meng *et al*., 2018). In addition, ethylene negatively regulates tomato sepal growth and cucumber fruit elongation (Li *et al*., 2017; Xin *et al*., 2019). Generally, ethylene inhibits organ growth and cell expansion/elongation through the auxin pathway (Hu *et al*., 2017). In contrast to the negative impacts of ethylene on the size of other organs, our previous studies show that ethylene positively regulates grain size in rice (Ma *et al*., 2013, 2018; Wuriyanghan *et al*., 2009; Yang *et al*., 2015). In this study, we further determine that the OsEIL1‐OsERF115‐grain‐size gene regulatory module is responsible for the positive effects of ethylene on grain size and weight. OsEIL1/MHZ6, the master TF of ethylene signalling pathway, has been shown to function as an important regulator of grain development (Yang *et al*., 2015). This work identifies *OsERF115* as a direct target of OsEIL1 in grain‐size regulation. More interestingly, among the *OsERF115*‐regulated grain‐size genes (Figure 3f), *OsBRI1*, *OsD11* and *OsSLG* are involved in BR biosynthesis, metabolism or signalling. Since BRs play a dominant role in modulating cell elongation and greatly affect organ size (Nolan *et al*., 2020), it appears that *OsERF115* connects the ethylene‐ and BR‐signalling pathways to regulate grain size, implying a novel mode of ethylene‐mediated organ size regulation via the BR pathway. In fact, the cross‐talk between ethylene and BR signalling pathways has been implicated in several biological processes such as root and shoot growth, apical hook formation and stress responses (Jiroutova *et al*., 2018). Further epistasis analysis of *OsERF115* and the BR‐related genes will facilitate a deep understanding on the ethylene–BR interplay during seed development.

Elite natural variations of agronomic traits have been successfully utilized in various breeding programmes. Sequence variation in the promoter region of a gene can lead to phenotypic differences caused by altered gene expression levels. Based on haplotype analysis of *OsERF115* from 2486 rice accessions, we find that SNPs located in the EBS motif of *OsERF115* promoter are significantly associated with the *OsERF115* expression levels and 1000‐grain weight. The result demonstrates that natural variations in *OsERF115* promoter contribute to grain‐size diversity in a wide range of rice varieties. This finding reveals a genetic basis for grain‐size variation in rice. Genomic selection of the Type1 haplotype with the intact EBS motif in rice breeding should help to increase grain size and yield.

Notably, OsERF115 orthologues are only found in grass species. Multiple sequence alignment reveals that the AP2/ERF domains are highly conserved among these orthologous proteins (Figure S12). Moreover, in accord with OsERF115 in rice, its orthologues from wheat, maize and sorghum all display transcriptional repression activity (Figure S13). These findings suggest that OsERF115 and its orthologues may have conserved function and play a unique role in grain development of grass species. The spikelet hull consisting of lemma and palea is a grass‐specific organ that largely affects grain size (Ren *et al*., 2019). During panicle development, the spikelets are tightly encased within leaf sheaths before heading, and the lemma and palea are also tightly closed before anthesis. Since internal ethylene content in plant tissues is largely affected by the gas diffusion rate (Cameron and Yang, 1982), it appears reasonable that due to restricted diffusion, ethylene gas can accumulate significantly in the spikelets. We speculate that *OsERF115* orthologous genes may emerge specifically in the grass species to mediate this entrapped ethylene gas to regulate grain development. On the other hand, the entrapped ethylene gas in spikelets may endow OsERF115 orthologues with a unique function in the grain‐size control in grass species. In fact, the significance of restricted ethylene diffusion has been reported in some environmental adaptation processes. For example, in deepwater rice plants under submerged conditions, ethylene content increases about 25‐fold due to impeded gas diffusion. The entrapped ethylene can trigger fast underwater internode elongation mediated by the *OsERF* genes *SNORKEL1*/2, thereby enabling the plants to escape from flooding submergence (Hattori *et al*., 2009). In addition, a recent work unravelled that the ethylene gas entrapped in roots by compacted soils can serve as a critical signal for plants to sense soil compaction (Pandey *et al*., 2021). Overall, it would be interesting to address the regulatory roles of *OsERF115* orthologues in the grain development of other cereal crops.

## Materials and methods

### Plant growth conditions and phenotypic analysis

Rice plants were grown in paddy fields in the experimental stations of USTB in Beijing from May to October, Hunan Province from July to November, and Hainan Province from November to next April. *Nipponbare* was used as WT unless indicated otherwise. The T_2_ or T_3_ transgenic rice plants were used for phenotypic analysis. At least 30 spikelet hulls were harvested at different stages before heading and used for measurements of length and width. More than 30 fully filled grains were used to measure the length, width and thickness of grains and 1000‐grain weight. Grain‐filling rates were investigated by measuring the dry weight of 20 caryopses sampled at 1, 7, 14, 21, 28 and 35 DAP.

### Vector construction and rice transformation

The *OsERF115* coding sequence (570 bp) was PCR‐amplified from *Nipponbare* and cloned into *Xba* I/*Sal* I‐digested *pCAMBIA2300‐35S* and *Kpn* I/*Sal* I‐digested *pCAMBIA2300‐35S‐GFP* vectors by homologous recombination to generate *OsERF115‐OX* and *35S:OsERF115‐GFP* constructs respectively. For knockout vectors of *OsERF115*, the knockout targets were screened by the web‐based tool CRISPR‐P 2.0 (http://cbi.hzau.edu.cn/cgi‐bin/CRISPR) to prevent potential off‐target sites (Liu *et al*., 2017). The CRISPR/Cas9 plasmids (*OsERF115‐Cas9‐1* and *OsERF115‐Cas9‐2*) were constructed by inserting the different knockout targets into *pHUE411* (Xing *et al*., 2014). All the vectors were introduced into *Agrobacterium tumefacines* EHA105 using the freeze‐thaw method, and the transformed strains were used to conduct rice genetic transformation as described previously (Toki *et al*., 2006). More than 30 independent transgenic lines (T_0_) were generated for each construct. The overexpression transgenic lines were selected by hygromycin and confirmed by PCR. The expression of *OsERF115* was analysed by RT‐qPCR with primers specific for the vector sequence named OX‐F/R to avoid detection of the endogenous transcript. Homozygous transgenic lines were obtained based on the hygromycin resistance of T_2_ plants. To examine the mutation sites of the knockout lines, we extracted the genomic DNA from the transgenic plants for PCR amplification of the designed target sites, and the various types of *OsERF115* were confirmed by sequencing. The primers used for plasmids construction are listed in Table S6.

### Droplet digital PCR

Hi‐DNAsecure Plant Kit (TIANGEN, PD350, Beijing, China) was used to extract and purify the genomic DNA from the young leaves of the overexpression transgenic plants according to the manufacturer’s instructions. DNA samples were quantified using a Nano‐Drop 2000 (Thermo, ND‐2000C, Waltham, Massachusetts, the United States) and diluted to 25 ng/μL as the digital PCR templates. The ddPCR was conducted on the QX200™ Droplet Digital™ PCR platform (Bio‐Rad) as described previously (Li *et al*., 2020). The specific primers/probes of *CaMV35S* promoter (*35S*), *HPT II* gene and the rice reference gene *PLD* were added into the initial PCR mixture. The water‐in‐oil emulsions were generated in a QX200™ Droplet Generator (Bio‐Rad, DG8™) by using an eight‐channel disposable droplet generator cartridge. Then, the mixture was transferred into a 96‐well plate to perform PCR with 40 cycles. The data acquisition and analysis were performed using the QX200™ Droplet Reader (Bio‐Rad) and the calculation of target gene copy number per reaction was conducted using QuantaSoft™ Version 1.7.4.0917 (Bio‐Rad). The estimated copy numbers in the overexpression transgenic event were calculated by dividing the *35S* or *HPT II* copy number by the *PLD* copy number. The non‐genetically modified rice DNA template was set as the negative control to monitor for false‐positive reactions. All experiments were performed in three parallel panels. The primers/probes used in ddPCR analysis are listed in Table S6.

### Real‐Time Quantitative PCR (RT‐qPCR) analysis

Total RNA was isolated from various rice tissues using Trizol Reagent (Invitrogen, Carlsbad, California, the United States). RNA samples were quantified using a Nano‐Drop 2000 (Thermo, ND‐2000C). The first‐strand cDNA was synthesized with 2 µg of total RNA by using 5X All‐In‐One RT MasterMix kit (abm, G492, Richmond, British Columbia, Canada) according to the instructions from the supplier. RT‐qPCR assays were performed on the QuantStudio 5 Real‐Time PCR System (ABI) using TB Green™ Premix Ex Taq™ (TaKaRa, RR820A, Kyoto, Japan). *OsActin1* was used as the as internal control. The data were calculated by the $2^{‐\Delta \Delta C_{t}}$method. All experiments were conducted with three independent biological replicates and three technical replicates. The primers used in RT‐qPCR analysis are listed in Table S6.

### Subcellular localization analysis

The plasmids of *35S:OsERF115‐GFP* and *35S:GFP* were co‐transformed with a nuclear marker *mCherry‐Ghd7* vector (Xue *et al*., 2008) into rice leaf protoplasts isolated from 7‐days‐old etiolated seedlings. After 16 h of incubation at 28 °C, the images of GFP signal were taken using a confocal microscope (Zeiss, LSM780, Oberkochen, Germany). The excitation/emission wavelengths were set at 488 nm/500–530 nm for GFP and 561 nm/582–639 nm for mCherry.

### Cytological observation and microscopy

For histological analysis, the fresh spikelet hulls were collected before heading and the developing endosperms were sampled at 7 DAP from uniformly grown WT, *OsERF115‐OX* and *OsERF115‐Cas9* transgenic plants.

For histological assays of spikelet hulls, the materials were fixed in FAA (3.7% formaldehyde, 50% ethanol and 5.0% glacial acetic acid for 16 h), dehydrated in an ethanol series, and then embedded in Paraplast Plus (Sigma, P3683, Saint Louis, Missouri, the United States). Tissues were cut into 8‐μm thick with a rotary microtome (KEDEE, KD‐2258S, Zhejiang, China), dewaxed in xylene, gradually rehydrated in a graded ethanol series and, finally, stained with 0.05% Toluidine Blue for light microscopy.

For transverse section assays of endosperms, the 7 DAP caryopsis samples were fixed in FAA, and then dehydrated in a graded ethanol series. Fixed samples were embedded in Spurr resin (SPI‐Chem™, GS02680) and cut into 2‐μm‐thick sections using a Leica RM2265 rotary microtome and stained with 0.05% Toluidine Blue. Finally, cross‐sections images were collected using the Olympus BX‐53 microscope, Olympus Corporation, Tokyo, Japan.

For SEM analysis, the mature grains were critical‐point dried and coated with gold: palladium (3:2) by an ion sputter coater (JEOL, JFC‐1100, Welwyn Garden City, the United Kingdom). SEM images were observed with a HITACHI S‐3400N scanning electron microscope, Tokyo, Japan. Cell number and cell area in the outer parenchyma layer of the spikelet hulls were measured using ImageJ, Bethesda, Maryland, the United States and Adobe Photoshop CS2 software, San Jose, California, the United States.

### Ethylene treatment

Young panicles were treated with ethylene as previously described (Ma and Zhang, 2017; Zhu *et al*., 2011) with minor modifications. Briefly, panicles (10 cm) were cut from uniformly grown WT and *Osein2* plants before heading and immediately placed in test tubes containing ½ MS liquid medium, keeping only the panicle necks submerged. The test tubes were then placed in 4 airtight plastic containers and incubated at 28 °C in the dark for 24 h. Into the first three containers, we injected equal volumes of ethylene gas (final concentration is 100 ppm), but at different time points of the 24‐h incubation: 0, 12, 18 h. Corresponding sample labels are 24‐, 12‐ and 6‐h treatment. The fourth container received no ethylene and was used as air control (sample label: 0 h treatment). At the end of the incubation, all samples were collected and frozen in liquid nitrogen. Samples were kept at −80 °C until used for RNA extraction.

### Transient dual‐luciferase assay in rice protoplasts

For transcriptional activity analysis of OsERF115, the coding region of *OsERF115* was cloned into *Bam* HI/*Sal* I‐digested *pRTBD*, which contains the GAL4 binding domain and fused with the GAL4‐BD‐VP16 in the *pRTBD‐VP16* vectors to generate *BD‐OsERF115* and *VP16‐OsERF115* used as effector plasmids. The 5x GAL4‐TATA:LUC vector which contains the GAL4 binding motif and coding region of luciferase was used as reporter plasmid.

For transcriptional activation assays of *OsERF115* by OsEIL1 or OsEIL2, the GAL4‐BD fragment was cut out from the *pRTBD* vector by using *Sac* I and *Xba* I to generate *35S‐Ω‐Vector*. The coding regions of *OsEIL1* or *OsEIL2* were cloned into *Xba* I/*Bam* HI‐digested *35S‐Ω‐Vector* to generate *35S‐Ω‐OsEIL1* and *35S‐Ω‐OsEIL2* was used as effector plasmids. The *35S‐Ω‐Vector* was co‐transformed with each reporter plasmids into the protoplasts as the negative control to diminish the background difference. The promoter region of *OsERF115* (1500 bp before ATG) was cloned into *Kpn* I/*Bam* HI‐digested pEASY‐LUC vector to generate *proOsERF115:LUC* used as the reporter plasmid.

For transcriptional activation assays of the downstream genes by OsERF115, the coding region of *OsERF115* was cloned into *Sac* I /*Xba* I‐digested *35S‐Ω‐Vector* to generate 35S‐Ω‐OsERF115 used as effector plasmid. Take *OsGW2* and *OsGS6* as examples; the promoter regions of *OsGW2* (2465 bp before ATG) and *OsGS6* (2007 bp before ATG) were cloned into *Kpn* I/*Bam* HI‐digested pEASY‐LUC vector to generate *proOsGW2:LUC* and *proOsGS6:LUC* plasmids used as reporters. The *35S‐Ω‐Vector* was also used as the negative control plasmid.

For all dual‐luciferase assays, the *proAtUbiquitin3: REN* was used as internal control plasmid co‐transformed with the effector and reporter plasmids into rice protoplasts isolated from 7‐days‐old etiolated seedlings. After a 16‐h incubation at 28 °C in the dark, the protoplasts were harvested and lysed and the relative LUC activity (LUC/REN) was determined using a Dual‐Luciferase Reporter Assay System (Promega, E1960, Madison, Wisconsin, the United States) according to the manufacturer’s instructions. Each assay was determined in three independent biological replicates. Primers used in transient dual‐luciferase assay are listed in Table S6.

### RNA‐sequencing analysis

Young panicles (approximate 5 and 10 cm in length) and caryopses (2 DAP and 7 DAP) were collected from the WT and *OsERF115‐*overexpression line (*OX5‐1*) for total RNA preparation. RNA sequencing libraries were prepared from the spikelet hull and caryopsis samples with two biological replicates. The library products were sequenced using Illumina Hiseq 4000 platform. The filtered clean reads were mapped to the rice Nipponbare reference genome (http://rice.plantbiology.msu.edu/) using HISAT2 with default parameters (Kim *et al*., 2019). Gene expression levels were calculated and normalized into RPKM (reads per kb per million mapped reads) values using DESeq2 (Love *et al*., 2014). Differentially expressed genes (DEGs) were identified with expression‐level fold changes >2 and false discovery rate (FDR) <0.05 between WT and *OX5‐1*. The agriGO (v2.0) online service (Tian *et al*., 2017) was used for gene ontology enrichment analysis of the DEGs.

### EMSA

The EMSA assay of direct binding of OsEIL1 to the promoter of *OsERF115* was performed as described previously (Yang *et al*., 2015). Briefly, the DNA fragment corresponding to the N‐terminal region (amino acids 1–350) of *OsEIL1* was fused with GST‐coding sequence and inserted into the *pGEX‐4T* vector to generate expression vector *pGEX‐OsEIL1N*. The plasmid was transformed into *E. coli* BL21 cells and the fusion proteins were induced with addition of 0.2 mmIPTG at 16 °C overnight. The cells were harvested and lysed using an ultrasonic cell disruptor (Diagenode, Liege, Belgium, BioruptorPlus, Liege, Belgium). The recombinant proteins were purified with glutathione agarose (Thermo, 16100) from cellular lysates, and eluted using a buffer containing reduced glutathione. The biotin‐labelled probes and competitors of *OsERF115* promoter were generated by annealing of biotinylated and unlabelled complementary primer pairs respectively. The EMSA reaction solutions with EMSA/Gel‐Shift Binding Buffer (Beyotime, GS005, Shanghai, China) were incubated for 30 min at room temperature. The reaction products were analysed on native polyacrylamide gels (6%) and transferred to a nylon membrane (GE, Pittsburgh, Pennsylvania, the United States). The biotin end‐labelled DNA was detected using the Chemiluminescent Nucleic Acid Detection Module (Thermo, 89880) on a nylon membrane. To determine the specificity of the DNA‐protein interaction, the unlabelled DNA probe with different fold were used to detect the specific interaction of the DNA–protein and the GST protein was used as a negative control.

For direct binding of *OsERF115* to the promoters of *OsGW2* and *OsGS6*, the recombinant MBP‐OsERF115 protein was expressed and purified from *E. coli* BL21. To construct plasmids for the expression of MBP‐OsERF115, the coding region of *OsERF115* was inserted into the *pMCSG7* vector by homologous recombination. The fusion proteins were induced by adding IPTG to the culture medium. After harvesting and lysing the cells, the recombinant proteins were affinity‐purified with Ni‐NTA resin (Bio‐red, 30210) and desalted with PD‐10 Desalting Columns (GE Healthcare, 17‐0851‐01) according to the manufacturer’s instructions. The probe labelling and the EMSA reactions were performed as described above. The MBP protein was used as a negative control. Primers and probes used in EMSA assay are listed in Table S6.

### ChIP‐qPCR

ChIP assay was performed as described previously (Yamaguchi *et al*., 2014) with minor modifications. Briefly, a total of 1 g rice tissues were cross‐linked with 1% formaldehyde for 30 min. The cells were lysed and the nuclei‐containing fractions were isolated. The chromatins were sheared using a sonicator (Bioruptor, UCD‐300) to the size range of 200–1000 bp; 2% volume of the supernatants was recovered as input. The chromatin suspensions were immunoprecipitated with protein G magnetic beads (Invitrogen, 78608) conjugated to anti‐Flag antibody (MBL, M185‐3, Kyoto, Japan), or GFP‐Trap Magnetic Agarose beads (Chromotek, gtma‐20, Planegg‐Martinsried, Germany), and the WT and 35S:GFP transgenic rice plants were as negative controls. The precipitated DNA fragments were recovered and quantified by real‐time PCR analyses as described above. The % input of the target genes were determined by the cycle threshold in IP sample relative to that of the input sample using the formula 2^‐[cq IP‐(cqinput‐5.64)]. Relative enrichment of a gene was normalized to the respective negative controls. Primers and probes used in ChIP‐qPCR are listed in Table S6.

### Haplotype analysis of *OsERF115* promoter

The SNPs in the 1.5‐kb promoter regions of *OsERF115* from 3010 rice varieties and the geographical information of cultivated rice were downloaded from the Rice Functional Genomics (Wang *et al*., 2018) (RFGB, http://www.rmbreeding.cn/Snp3k). The ggmap was used to mark geographical information on map to observe distribution characteristics of two alleles (Kahle & Wickham, 2013). After eliminating the heterozygotes and the haplotype with less than five varieties, 2486 varieties were retained and divided into 9 haplotypes. A local association test of variations in *OsERF115* promoter region with 1000‐grain weight was performed using Tassel 3.0 software with MLM Q + K model (Bradbury *et al*., 2007; Yu *et al*., 2006). To provide experimental evidence for haplotype analysis, we sequenced the promoter regions of *OsERF115* from 11 *indica* cultivars (Kasalath, 93‐11, Minghui63, Zhefu802, Zhuo21S, 20508, Wushansimiao, Fuoshan385, Meixiangzhan‐2, Yanxian156 and 2DC783), which were available in our laboratory and found that the sequences can be classified into four haplotypes of Types 4–7. The promoters of Types 4–7 and a series of mutated promoters were cloned into *Kpn* I/*Bam* HI‐digested *pEASY‐LUC* vector for promoter activity analysis as described above. The primers used are listed in Table S6.

### Phylogenetic analysis of *OsERF115*

For identifying orthologues of OsERF115, the BLAST search was performed on using the OsERF115 protein sequence in multiple published databases, including Gramene (http://www.gramene.org/), Phytozome (https://phytozome.jgi.doe.gov/), NCBI (https://www.ncbi.nlm.nih.gov/) and The *Arabidopsis* Information Resource (https://www.arabidopsis.org/). A neighbour‐joining tree based on the OsERF115 protein sequences in grass species was constructed using DNAMAN v8 software (Lynnon Biosoft, San Ramon, California, the United States). The coding sequences of orthologues from wheat (Chinese Spring), maize (B73) and sorghum (T × 430) were cloned into *EcoR* I‐digested *pRTBD‐VP16* vector and transformed into maize protoplasts for transcriptional activity analysis as described previously (An *et al*., 2020). All experiments were determined in three independent biological samples, each consisting of three technical repetitions. The primers used are listed in Table S6.

## Conflict of interest

The authors declare no conflict of interest.

## Author contributions

B.M., X. Wei and X. Wan designed research; C.L., T.M., D. Yuan, Y.Z., Y.L., M.D., D. Yu, Y.J., X.B., S.L., D.L. and X.L. performed research; X. Wei., Z.L., Z.D., Y.W., Q.H., J.Z., J.L. and S.C. analysed data; Z.L., and W.W. provided 1000‐grain weight of 3000 rice accessions; and B.M., C.L., X. Wei and X. Wan wrote and revised the paper.

## Supporting information


**Figure S1** Identification of seed‐specific *OsERF* genes.
**Figure S2** Phenotypic analysis of *OsERF115‐OX* lines.
**Figure S3** Phenotypic analysis of *OsERF115*‐OX lines grown in Changshan, Hunan province, China.
**Figure S4** Overexpression of *OsERF115* in *indica* rice variety Kasalath (Kas).
**Figure S5** Gene structure and sequence analysis of *OsERF115* mutations generated by a CRISPR/Cas9 system.
**Figure S6** Cytological observation of spikelet hulls and developing endosperms of WT, *OX5‐1* and *Cas9‐2*.
**Figure S7** Transcriptomics analysis of WT and one *OsERF115* overexpressing line (*OX5‐1*).
**Figure S8** OsERF15 indirectly induced *OspPLAIIα*, *OsPUP7* and the genes of spikelet hull growth and endosperm development.
**Figure S9** Functional characterization of *OsERF115‐GFP* fusion gene.
**Figure S10** OsEIL1 acts in a common pathway with OsERF115 to regulate grain size and weight.
**Figure S11** Native promoter activity analysis of the *OsERF115* promoters with different variations and site‐directed mutations at the two SNPs activated in rice protoplasts.
**Figure S12** Alignment of the AP2/ERF domains in OsERF115 orthologous proteins. Multiple amino acid sequences were aligned using DNAMAN v8 (Lynnon Biosoft) with the default parameters.
**Figure S13** Transactivation assay of OsERF115 orthologues from wheat, maize and sorghum in maize protoplasts using dual‐luciferase reporter system.

**Table S1** Protein sequence identity between OsERF115 orthologues.
**Table S2** 186 DEGs between WT and *OsERF115‐OX* line in 5‐cm rice panicles.
**Table S3** 604 DEGs between WT and *OsERF115‐OX* line in 10‐cm rice panicles.
**Table S4** 2392 DEGs between WT and *OsERF115‐OX* line in 2 DAP rice caryopses.
**Table S5** 2774 DEGs between WT and *OsERF115‐OX* line in 10 DAP rice caryopses.
**Table S6** List of primers used in this study.

## Data Availability

All data are presented as figures. Raw data can be provided if needed. A complete set of RNA‐seq raw data has been deposited in the National Center for Biotechnology Information (NCBI) Gene Expression Omnibus (GEO) database (BioProject ID: PRJNA701677).
