# Supplementary material for: The OsEIL1‐OsERF115‐target gene regulatory module controls grain size and weight in rice
Source: Plant Biotechnol J. 2022 May 6;20(8):1470–86. doi: 10.1111/pbi.13825 (PMC9342608; doi:10.1111/pbi.13825)
Supplement: Supplementary file 1 — Figure S1 Identification of seed‐specific OsERF genes. Figure S2 Phenotypic analysis of OsERF115‐OX lines. Figure S3 Phenotypic analysis of OsERF115‐OX lines grown in Changshan, Hunan province, China. Figure S4 Overexpression of OsERF115 in indica rice variety Kasalath (Kas). Figure S5 Gene structure and sequence analysis of OsERF115 mutations generated by a CRISPR/Cas9 system. Figure S6 Cytological observation of spikelet hulls and developing endosperms of WT, OX5‐1 and Cas9‐2. Figure S7 Transcriptomics analysis of WT and one OsERF115 overexpressing line (OX5‐1). Figure S8 OsERF15 indirectly induced OspPLAIIα, OsPUP7 and the genes of spikelet hull growth and endosperm development. Figure S9 Functional characterization of OsERF115‐GFP fusion gene. Figure S10 OsEIL1 acts in a common pathway with OsERF115 to regulate grain size and weight. Figure S11 Native promoter activity analysis of the OsERF115 promoters with different variations and site‐directed mutations at the two SNPs activated in rice protoplasts. Figure S12 Alignment of the AP2/ERF domains in OsERF115 orthologous proteins. Multiple amino acid sequences were aligned using DNAMAN v8 (Lynnon Biosoft) with the default parameters. Figure S13 Transactivation assay of OsERF115 orthologues from wheat, maize and sorghum in maize protoplasts using dual‐luciferase reporter system. [file PBI-20-1470-s001.docx]

**Supplemental Figures 1-13**

**The OsEIL1-OsERF115-target gene regulatory module controls grain size and weight in rice**

Chang Liu^1,3,9^, Tian Ma^2,9^, Dingyang Yuan^4,5,9^, Yang Zhou^6^, Yan Long^1,3^, Ziwen Li^1,3^, Zhenying Dong^1,3^, Meijuan Duan^5^, Dong Yu^5^, Yizhi Jing^1^, Xiaoyue Bai^1^, Yanbo Wang^1^, Quancan Hou^1,3^, Shuangshuang Liu^1,3^, Jin-Song Zhang^6^, Shou-Yi Chen^6^, Dayong Li^7^, Xue Liu^7^, Zhikang Li^8^, Wensheng Wang^8^, Jinping Li^3^, Xun Wei^1,3*^, Biao Ma^2 *^ and Xiangyuan Wan^1,3*^

^1^ Zhongzhi International Institute of Agricultural Biosciences, Shunde Graduate School, Research Center of Biology and Agriculture, University of Science and Technology Beijing, 100024 Beijing, China;

^2^College of Agriculture, South China Agricultural University, Guangdong Laboratory for Lingnan Modern Agriculture, 510642, Guangzhou, China;

^3^ Beijing Engineering Laboratory of Main Crop Bio-Tech Breeding, Beijing International Science and Technology Cooperation Base of Bio-Tech Breeding, Beijing Solidwill Sci-Tech Co. Ltd., 100192 Beijing, China;

^4^ State Key Laboratory of Hybrid Rice, Hunan Hybrid Rice Research Centre, 410125 Changsha, China;

^5^ College of Agronomy, Hunan Agricultural University, 410128 Changsha, China;

^6^ State Key Laboratory of Plant Genomics, Institute of Genetics and Developmental Biology, Chinese Academy of Sciences, 100101 Beijing, China;

^7^ National Engineering Research Center for Vegetables, Beijing Vegetable Research Center, Beijing Academy of Agriculture and Forestry Science, Beijing, 100097, China;

^8^ Institute of Crop Sciences, Chinese Academy of Agricultural Sciences, Beijing, 100081, China.

^9^ These authors contributed equally to this article.

*Correspondence: Xiangyuan Wan (wanxiangyuan@ustb.edu.cn), Biao Ma

mabiao@scau.edu.cn, or Xun Wei (weixun@ustb.edu.cn)

**Thirteen Supplemental Figures and Six Supplemental Tables**


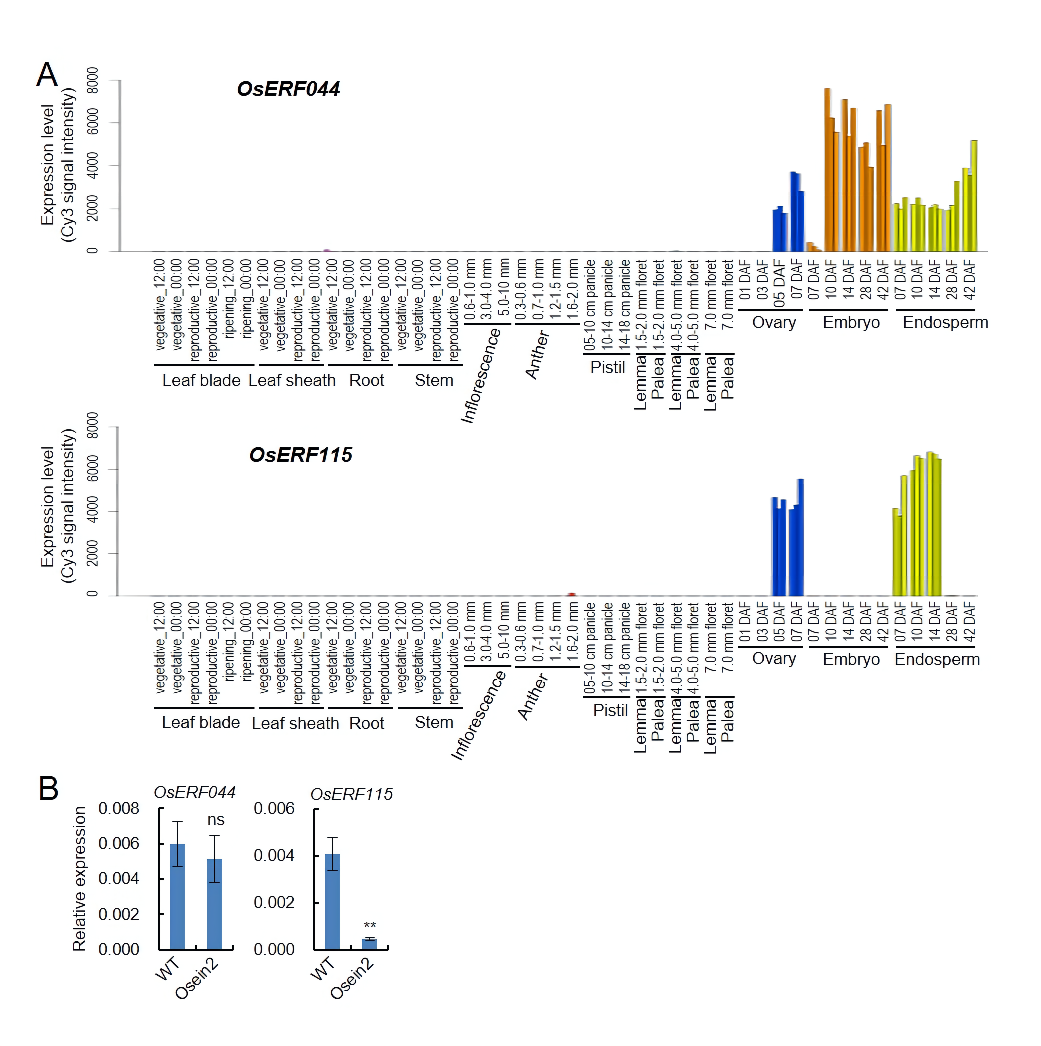


**Figure S1. Identification of seed-specific *OsERF* genes.**

**A)** Spatiotemporal expression patterns of *OsERF044* and *OsERF115* based on the data from Rice Expression Profile Database (RiceXPro) (<http://ricexpro.dna.affrc.go.jp/>). **B)** Expression levels of *OsERF044* and *OsERF115* in WT (Nipponbare) and *Osein2* mutant analyzed by RT-qPCR. Data are means ± SD, n = 3. ***P* < 0.01, Student’s *t* test. ns, no significance.


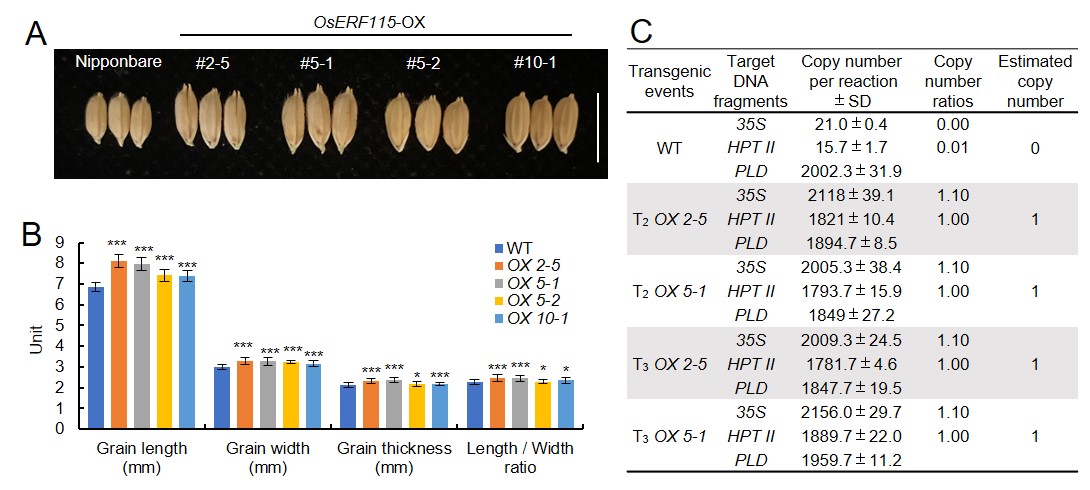


**Figure S2. Phenotypic analysis of *OsERF115*-OX lines.**

**A)** Grain phenotype of *OsERF115*-overexpressing lines in the japonica variety Nipponbare. Scale bar, 10 mm. **B)** Grain size and shape. Data are means ± SD, n = 50. **P* < 0.05, ****P* < 0.001, Student’s *t* test. **C)** T-DNA copy numbers as estimated by ddPCR analysis. *35S* and *HPT II* were used as the target DNA fragments, and *PLD* was used as the native control.


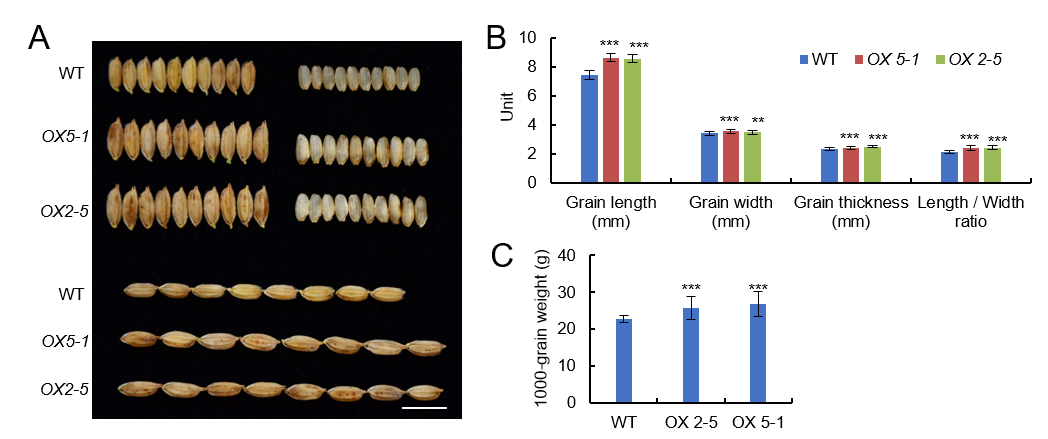


**Figure S3. Phenotypic analysis of *OsERF115*-OX lines grown in Changshan, Hunan province, China.**

**A)** Grain morphology of WT and *OsERF115-*OX mature grains. Scale bar, 10 mm. **B)** Grain size and shape. Data are means ± SD, n = 50. **C)** 1,000-grain weight. Data are means ± SD, n = 20. ***P* < 0.01, ****P* < 0.001, Student’s *t* test.


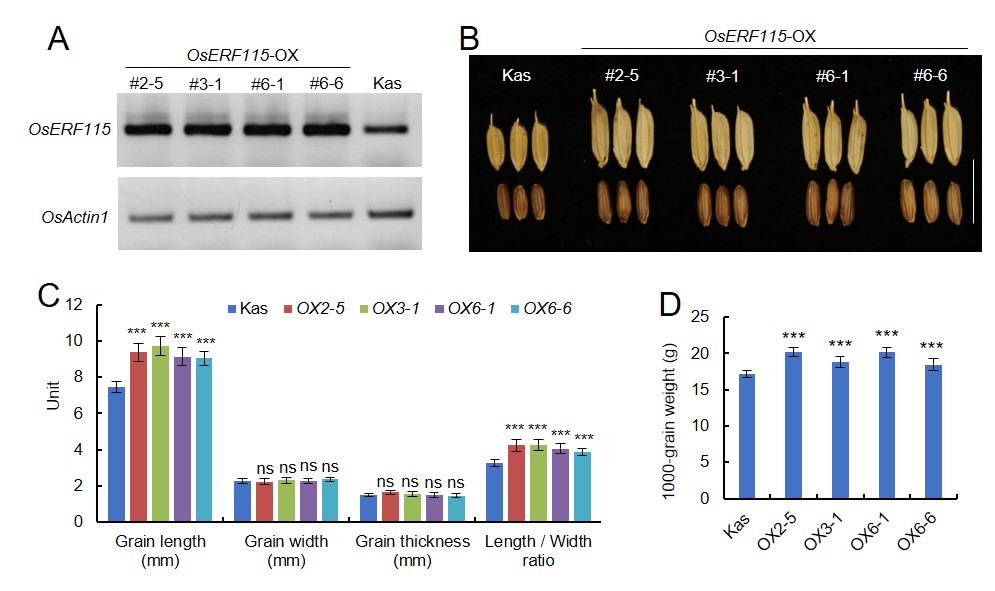


**Figure S4. Overexpression of *OsERF115* in *indica* rice variety Kasalath (Kas).**

**A)** Expression levels of *OsERF115* gene in four overexpressing lines detected by semiquantitative-PCR. *OsActin1* was used as an internal control. **B)** Grain morphology of Kas and four *OsERF115-*OX lines. Scale bar, 10 mm. **C)** Grain size and shape of Kas and four *OsERF115-*OX lines. Data are means ± SD, n = 50. **D)** 1,000-grain weight of Kas and four *OsERF115-*OX lines. Data are means ± SD, n = 10. ****P* < 0.001, Student’s *t* test. ns, no significance.


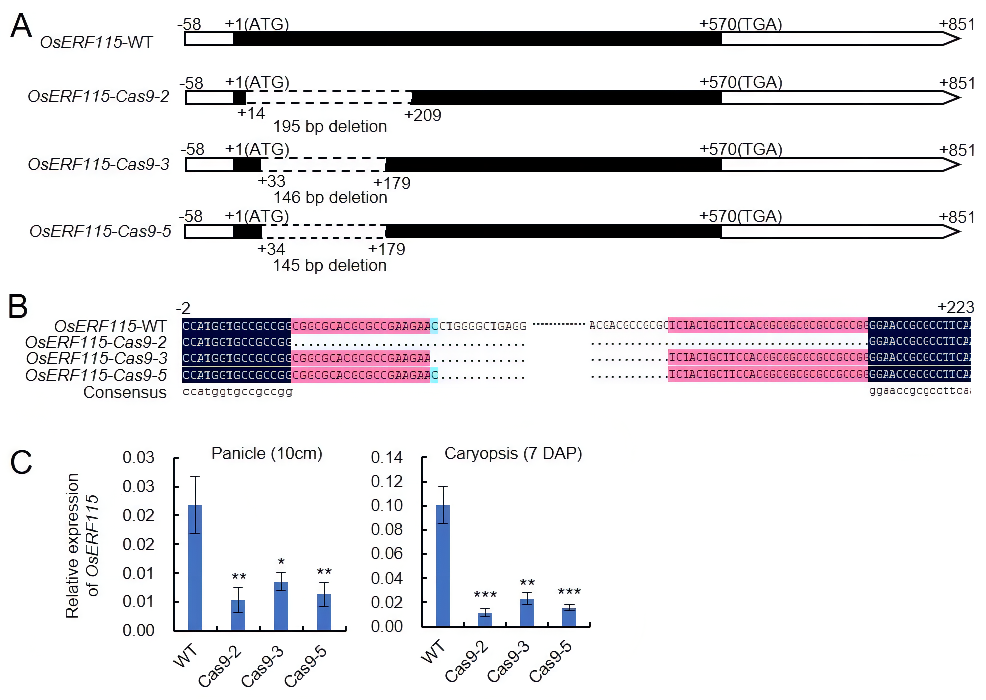


**Figure S5. Gene structure and sequence analysis of *OsERF115* mutations generated by a CRISPR/Cas9 system.**

**A)** Gene structure of *OsERF115* in WT and three types of knockout lines. **B)** DNA sequence alignment of the targeted mutation fragments between WT and three knockout lines. **C)** RT-qPCR analysis of *OsERF115* expression in its CRISPR/Cas9-edited lines. Data are means ± SD, n = 3. **P* < 0.05, ***P* < 0.01, ****P* < 0.001, Student’s *t* test.


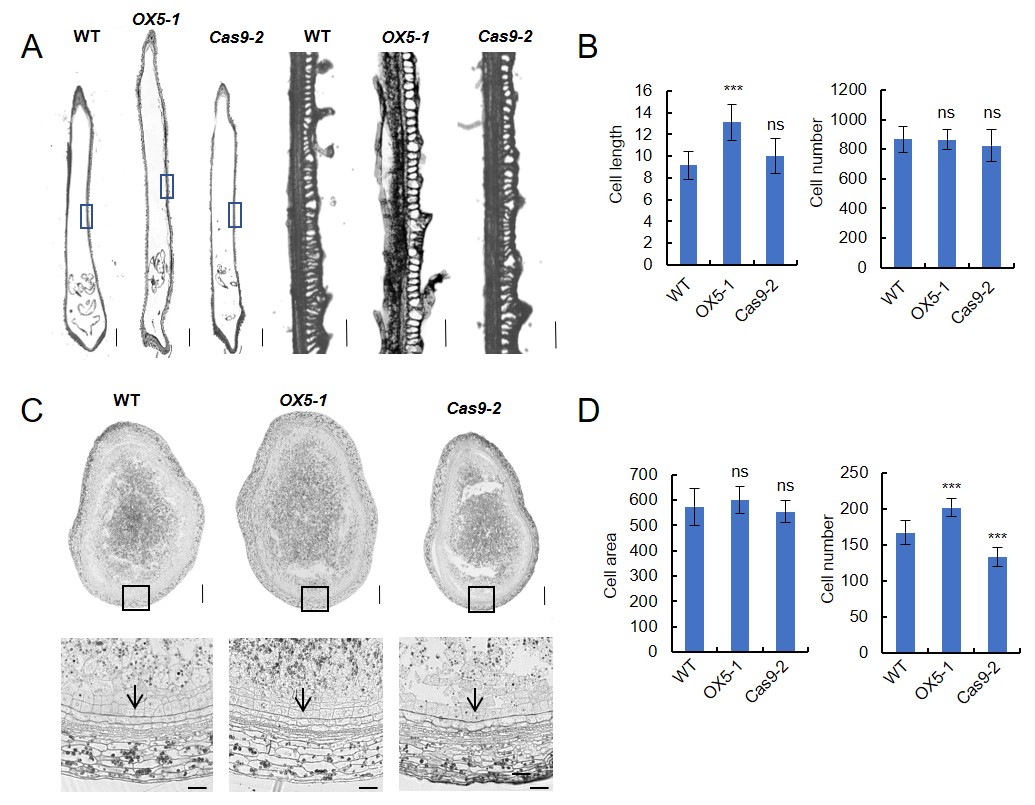


**Figure S6. Cytological observation of spikelet hulls and developing endosperms of WT, *OX5-1* and *Cas9-2.***

**A)** Longitudinal-section analysis of the spikelet hulls. Scale bar, 500 μm for whole spikelets longitudinal-section (left) and 50 μm for magnified views (right). **B)** Statistical data of the single epidermal cell length and total glume cell number. **C)** Cross-sections of developing endosperms of WT, *OX5-1* and *Cas9-2*. Scale bars, 100 μm for whole cross-sections endosperms (left), and 10 μm for magnified views (right). Arrows indicate the aleurone layer corresponding to the counted cells. **D)** Quantification of cell area and cell number in the aleurone layer. Data are means ± SD, n = 10. ****P* < 0.001, Student’s *t* test. ns, no significance.


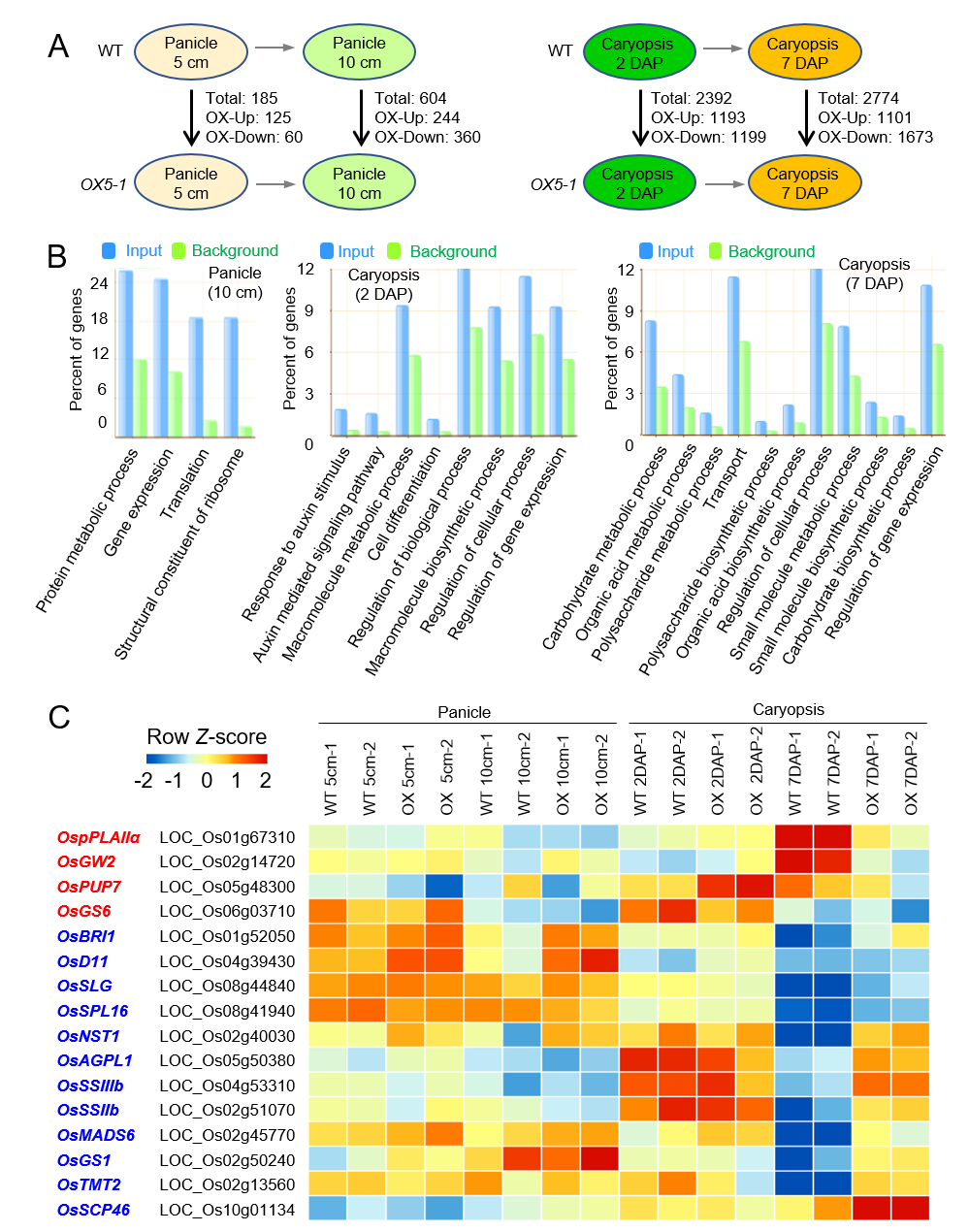


**Figure S7. Transcriptomics analysis of WT and one *OsERF115* overexpressing line (*OX5-1*).**

**A)** Differentially expressed genes (DEGs) between WT and *OX5-1* line. **B)** Gene ontology enrichment analysis of DEGs using the agriGO v2.0 program (<http://systemsbiology.cau.edu.cn/agriGOv2/>). **C)** Heatmap of expression levels of grain size-related genes in WT and *OX5-1* line. The red and blue gene names represent negative and positive regulators of grain size, respectively.


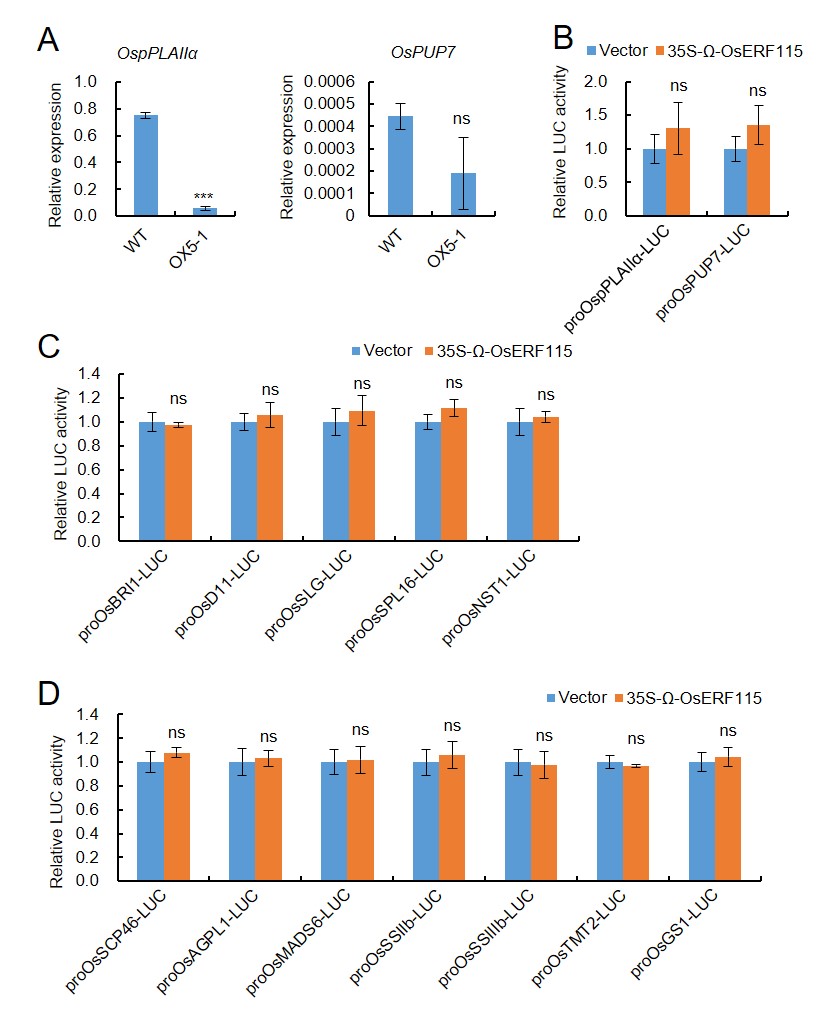


**Figure S8. OsERF115 indirectly regulates the expression of *OspPLAIIα*, *OsPUP7* and the genes involved in spikelet hull growth and endosperm development.**

**A)** Expression levels of *OspPLAIIα* and *OsPUP7* in 7 DAP caryopses of WT and *OX5-1* line analyzed by RT-qPCR. **B)** Dual-luciferase assay of promoter activities of *OspPLAIIα* and *OsPUP7* inhibited by OsERF115 in rice protoplasts. Data are means ± SD, n = 3. **C)** Dual-luciferase assay of promoter activity of grain length- and width-controlling genes regulated by OsERF115 in rice protoplasts. **D)** Dual-luciferase assay of promoter activity of grain filling-related genes regulated by OsERF115 in rice protoplasts. Data are means ± SD, n = 3. ****P* < 0.001, Student’s *t* test. ns, no significance.


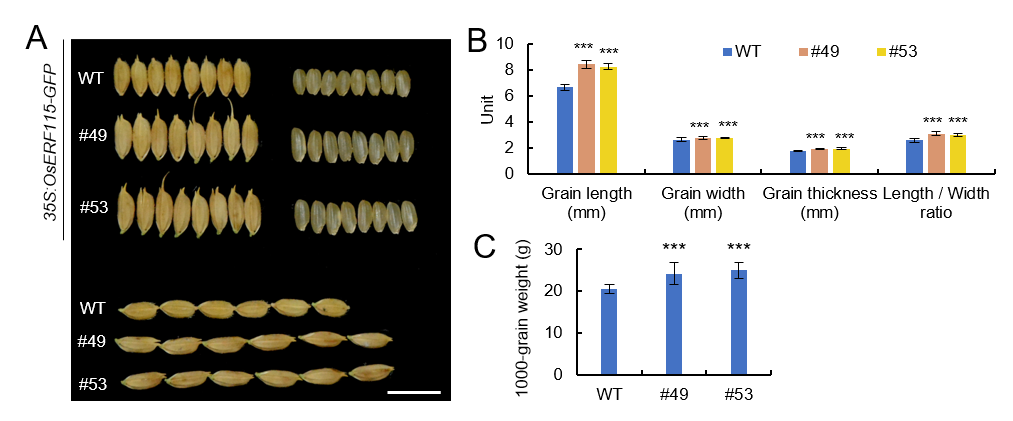


**Figure S9. Functional characterization of *OsERF115-GFP* fusion gene.**

**A)** Grain morphology of WT and *35S:OsERF115-GFP* transgenic rice plants. Scale bar, 10 mm. **B)** Statistical analysis of grain size and shape. Data are means ± SD, n = 50. **C)** Statistical analysis of 1,000-grain weight. Data are means ± SD, n = 10. ****P* < 0.001, Student’s *t*-test.


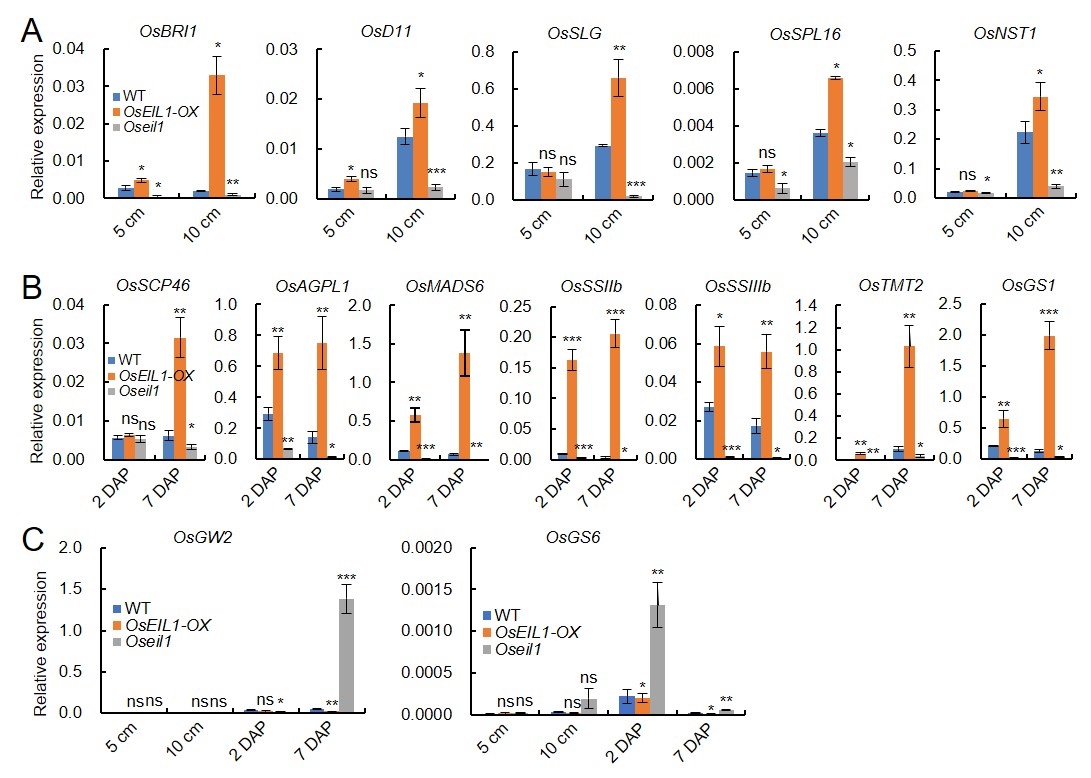


**Figure S10. Transcriptional regulation of grain size- and grain weight-related genes by OsEIL1.**

**A)** Expression levels of grain length- and width-controlling genes in young panicles of WT, *OsEIL1-OX* and *Oseil1* analyzed by RT-qPCR. **B)** Expression levels of grain filling-related genes in developing caryopses of WT, *OsEIL1-OX* and *Oseil1* analyzed by RT-qPCR. **C)** Expression levels of *OsGW2* and *OsGS6* in WT, *OsEIL1-OX* and *Oseil1* analyzed by RT-qPCR. Data are means ± SD, n = 3. **P* < 0.05, ***P* < 0.01, ****P* < 0.001, Student’s *t* test. ns, no significance.


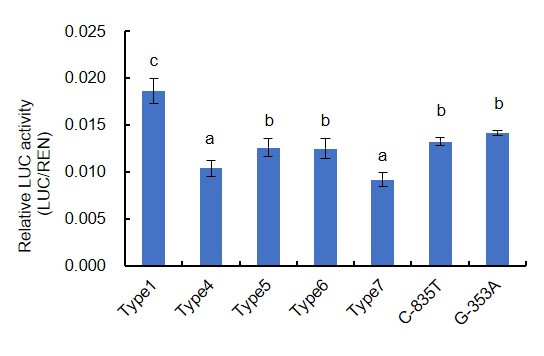


**Figure S11. Native promoter activity analysis of the *OsERF115* promoters with different variations and site-directed mutations at the two SNPs activated in rice protoplasts.**

Data are means ± SD, n = 3. Different letters indicate significant differences (*P* < 0.05, LSD test) in the multiple comparison.


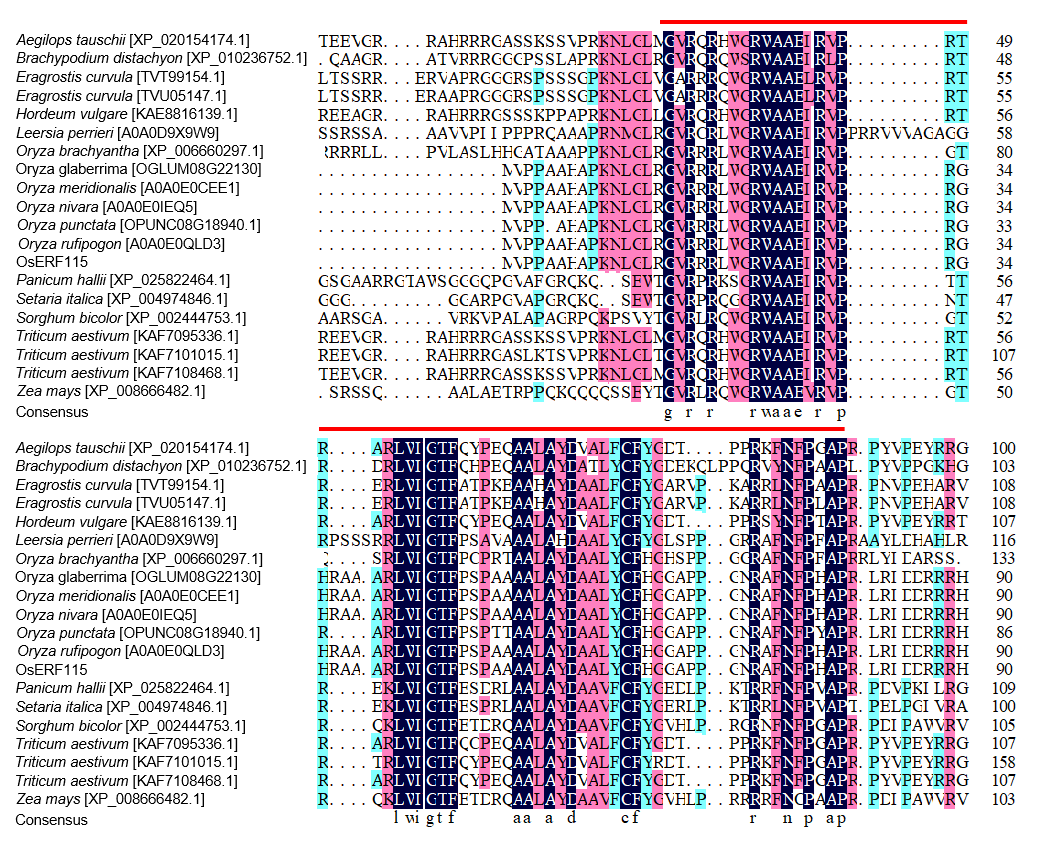


**Figure S12. Alignment of the AP2/ERF domains in OsERF115 orthologous proteins. Multiple amino acid sequences were aligned using DNAMAN v8 (Lynnon Biosoft) with the default parameters.**

Black shadings indicate identical residues; red and blue shadings indicate similar residues. The AP2/ERF domains are marked by red lines.


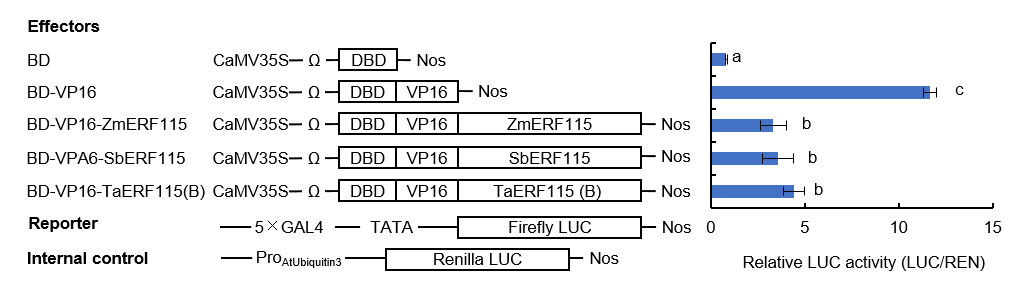


**Figure S13. Transactivation assay of OsERF115 orthologues from wheat, maize and sorghum in maize protoplasts using dual-luciferase reporter system.**

The main structure of vectors is shown at Middle. Data are means ± SD, n = 3. Different letters indicate significant differences (*P* < 0.01, LSD test) in the multiple comparison.
